# Small RNA Deep Sequencing Identifies a Unique miRNA Signature Released in Serum Exosomes in a Mouse Model of Sjögren's Syndrome

**DOI:** 10.3389/fimmu.2020.01475

**Published:** 2020-07-17

**Authors:** Shruti Singh Kakan, Srikanth R. Janga, Benjamin Cooperman, David W. Craig, Maria C. Edman, Curtis T. Okamoto, Sarah F. Hamm-Alvarez

**Affiliations:** ^1^Department of Pharmacology and Pharmaceutical Sciences, School of Pharmacy, University of Southern California, Los Angeles, CA, United States; ^2^Department of Ophthalmology, Keck School of Medicine, Roski Eye Institute, University of Southern California, Los Angeles, CA, United States; ^3^Department of Translational Genomics, Keck School of Medicine, University of Southern California, Los Angeles, CA, United States

**Keywords:** Sjögren's Syndrome, diagnostic miRNA biomarkers, extracellular vesicles, small-RNA sequencing, microRNA, piwi-RNA

## Abstract

Sjögren's Syndrome (SS) is an autoimmune disease characterized by lymphocytic infiltration and loss of function of moisture-producing exocrine glands as well as systemic inflammation. SS diagnosis is cumbersome, subjective and complicated by manifestation of symptoms that overlap with those of other rheumatic and ocular diseases. Definitive diagnosis averages 4–5 years and this delay may lead to irreversible tissue damage. Thus, there is an urgent need for diagnostic biomarkers for earlier detection of SS. Extracellular vesicles called exosomes carry functional small non-coding RNAs which play a critical role in maintaining cellular homeostasis via transcriptional and translational regulation of mRNA. Alterations in levels of specific exosomal miRNAs may be predictive of disease status. Here, we have assessed serum exosomal RNA using next generation sequencing in a discovery cohort of the NOD mouse, a model of early-intermediate SS, to identify dysregulated miRNAs that may be indicative of SS. We found five miRNAs upregulated in serum exosomes of NOD mice with an adjusted *p* < 0.05—miRNA-127-3p, miRNA-409-3p, miRNA-410-3p, miRNA-541-5p, and miRNA-540-5p. miRNAs 127-3p and 541-5p were also statistically significantly upregulated in a validation cohort of NOD mice. Pathway analysis and existing literature indicates that differential expression of these miRNAs may dysregulate pathways involved in inflammation. Future studies will apply these findings in a human cohort to understand how they are correlated with manifestations of SS as well as understanding their functional role in systemic autoimmunity specific to SS.

## Introduction

Sjögren's Syndrome (SS) is a chronic and systemic autoimmune disease marked by lymphocytic infiltration and loss of function of the body's moisture producing exocrine glands (e.g., lacrimal and salivary glands) as its defining manifestation. It is the second most common rheumatic autoimmune disease, affecting about 0.5–1% of the general population (1, 2). The progressive inflammation of lacrimal and salivary exocrine glands is associated with their loss of function, leading to debilitating dry eye and dry mouth, respectively (3). SS is associated with increased inflammation of internal organs including brain, lung and liver (4) as well as a 44-fold increased risk of developing B-cell lymphoma (5, 6). SS can occur in the absence of another autoimmune disease (primary SS) or concurrently with another autoimmune disease such as rheumatoid arthritis or systemic lupus erythematosus (secondary SS).

Diagnosis of SS relies on the weighted score obtained from a series of criteria established in 2016 by the American College of Rheumatology (ACR) in collaboration with the European League Against Rheumatism (EULAR) (7). These criteria include: (1) labial salivary gland biopsy showing focal lymphocytic sialadenitis with a focus score ≥1; (2) anti-SSA (Ro) positivity (serum autoantibody); (3) ocular surface staining score ≥ 5 (or van Bijsterveld score ≥ 4) on at least one eye; (4) a Schirmer's value (tear flow) ≤ 5 mm/5 min on at least one eye; and (5) an unstimulated whole saliva flow rate ≤ 0.1 ml/min. Although, commonly used for inclusion in clinical trials, these criteria are not always practically applicable for clinical diagnosis. In particular, the labial salivary gland biopsy is painful, impractical and error-prone (8). Furthermore, these criteria have been developed primarily for patients with primary SS, and are not extensively validated in the far greater numbers of patients suffering from secondary SS. Therefore, many patients experience a substantial delay in diagnosis while some are never formally diagnosed. This delay in diagnosis may also delay treatment with anti-inflammatory agents to the point when irreversible damage to exocrine glands and other internal organs may already have occurred (9). Hence, there is an urgent need for an early, sensitive and non-invasive diagnostic test for SS.

In this study we utilized the Non Obese Diabetic mouse model of SS. The NOD/Shi strain originated from inbreeding of the Cataract Shionogi (CTS) strain, based on elevated fasting blood glucose level in cataract-free mice for the development of a model for insulin dependent diabetes mellitus. It was later also shown to develop features of exocrinopathy consistent with SS (10). Despite limitations to any animal model, this model exhibits several features of SS in humans including reduced tear and salivary secretion (11, 12), alterations in tear and salivary composition (13–15), lymphocytic infiltration of lacrimal and salivary glands (dacryoadenitis and sialadenitis, respectively) (11, 12), and the presence of many of the serum autoantibodies that are often present in human patients, such as autoantibodies to Ro/SSA and La/SSB (16), the M3 muscarinic acetylcholine receptor (17, 18), salivary gland protein 1 (19), carbonic anhydrase 6 (19) and parotid secretory protein (PSP) (19). Finally, the lacrimal glands (LG) of these mice show characteristic changes in specific proteins involved in the secretory process of exocrine glands typical of SS patients (20, 21). We have previously demonstrated that tear biomarkers identified in this murine model are also identified in SS patients (13–15).

Although, SS is more prevalent in women than in men at a 9:1 ratio (3), only male NOD mice were used in this study, because the male mice have been extensively characterized to exhibit the features of autoimmune dacryoadenitis and systemic disease prior to the development of diabetes. Females of this strain instead develop autoimmune sialadenitis concurrent with diabetes (22), complicating interpretation of any results. Thus, use of male NOD mice allows us to avoid confounding effects associated with the concurrent development of diabetes. Our mice were chosen at the age just after lymphocytic infiltration of the LG is typically established (14-weeks), representing an early-intermediate stage disease model of autoimmune dacryoadenitis in SS. As the disease development in this strain is polygenetic, and many of the diabetes resistant sub-strains that have been developed as controls for studying diabetes development still develop autoimmune exocrinopathy (11, 23, 24) there has been a lack of closely related healthy control strains for studies of SS disease development and treatment. With this said, the Balb/c strain has been the most commonly used for studies of SS exocrinopathy by multiple groups beyond our own (25–28). Therefore, we considered it the most prudent choice for use as a control strain.

MicroRNAs (miRNA) are evolutionarily conserved short non-coding RNA that function in gene silencing and post-transcriptional gene regulation (29), regulating nearly 60% of messenger RNA (mRNA) (30). A single miRNA can target several mRNA and any given mRNA may be targeted by more than one miRNA. Cooperativity between a group of dysregulated miRNAs targeting one or more mRNAs of a given signaling pathway or cellular process may substantially upregulate or downregulate that pathway and lead to development and progression of disease. Indeed, miRNA dysregulation is associated with cancer (31), obesity (32), heart disease (33), kidney disease (34), and diseases of the nervous system (35). Their diagnostic potential has been explored with high fidelity in various cancers (36), as well as neurodegenerative (37), autoimmune (38), and metabolic diseases (39). Thus, assessing the level of expression of a panel of functional mature miRNAs can be diagnostic of a given disease. Compared to proteins, miRNAs have lower inter-individual variation and less sequence heterogeneity, allowing for high specificity as biomarkers (40). Further, as miRNAs are highly evolutionarily conserved in mammals, results from an animal model are typically readily applicable to human subjects (41).

miRNAs circulate in a stable, cell-free form in all biofluids and are particularly enriched in serum (42). Relative to other biofluids such as saliva, urine, and cerebrospinal fluid (CSF), serum has a higher concentration of miRNA (42, 43). Most extracellular miRNAs as well as other non-coding RNAs (ncRNA) can be found in exosomes which are nano-sized extracellular vesicles, generated as intraluminal vesicles in multivesicular bodies (MVBs). Exosomes are actively shed from nearly every cell type and engage in intercellular signaling. Literature suggests that aberrant exosome-based intercellular communication plays a role in infectious and inflammatory diseases and various cancers. Exosomes are an extremely reliable source of miRNA as their contents are resistant to degradation by nucleases such as RNase (36, 44–47). Moreover, exosomal miRNAs are relatively stable following storage at −80°C, have a very low inter-individual variance, and also exhibit a low intra-individual variance over time (48).

The goal of this study was to understand how changes in regulatory small RNAs in serum, particularly miRNAs, might have utility as a source of diagnostic biomarkers for SS. Here we identify a subset of dysregulated miRNAs that may be specific to SS. We identified 5 exosomal miRNAs that showed significant changes in concert with establishment of SS-like symptoms in NOD mice, representing putative biomarkers for early disease diagnosis. Additionally, we have also assessed differential expression of other small non-coding RNA such as piRNA, that protect the genome by silencing transposons.

## Materials and Methods

### Mice

Age-matched male NOD/ShiltJ (Stock No. 001976) and BALB/cJ (Stock No. 000651) mice were purchased from Jackson Laboratories (Bar Harbor, ME) and housed with a 12 h light, 12 h dark cycle with *ad libitum* access to food and water until 14-weeks of age, when SS-like ocular symptoms are established in the NOD strain. All procedures performed on the mice were in accordance with protocols approved by the University of Southern California's Institutional Animal Care and Use Committee (IACUC) and the Guide for Care and Use of Laboratory Animals 8th edition (49).

### LG Histology and Quantitative Analysis of Lymphocytic Infiltration

Lymphocytic infiltration in mouse lacrimal glands, indicative of autoimmune dacryoadenitis, was confirmed and quantified with hematoxylin & eosin staining as described (50). Briefly, lacrimal glands from NOD and BALB/c mice were fixed in 10% NBF (Richard-Allan Scientific, Kalamazoo, MI), fixed in paraffin, then cut into 5 μ horizontal sections and stained with hematoxylin and eosin. Sections were imaged using an Aperio Digital ScanScope (Leica Biosystems Inc., Buffalo Grove, IL) using the 40x objective lens. The percentage of lymphocytic infiltration in the tissue was determined by calculating the area of infiltrates manually using ImageJ (National Institutes of Health, http://imagej.nih.gov/ij). Data were analyzed by GraphPad Prism using one-way non-parametric ANOVA (Kruskal-Wallis).

### Isolation of Serum Exosomes

Mice were anesthetized by intraperitoneal injection with ketamine/xylazine (60–70 mg + 5–10 mg/kg, respectively), and blood was collected by cardiac puncture using a 1 mL syringe (BD Biosciences, San Jose, CA) into MiniCollect 0.8 mL gold cap Z Serum Separator tubes (Greiner Bio-One, Kremsmünster, Austria). Thereafter the mice were euthanized by cervical dislocation. Blood was allowed to clot for 20 min at room temperature followed by centrifugation at 4°C, 2,000 × g for 15 min. Serum was collected and spun at 2,000 × g for 20 min at 4°C to pellet cellular debris. The supernatant was collected and spun at 12,000 × g for 45 min to remove microvesicles. Approximately 2000 μL of pooled supernatant from 5 mice was concentrated using 10 kDa Millipore Amicon Ultra concentrators (Burlington MA) to 500 μL and then loaded on an equilibrated iZON qEV original size exclusion column (Christchurch, New Zealand). Fractions 7–9 containing 1.5 mL of exosomes were collected and concentrated by 10-fold using 100 kDa concentrators (MilliporeSigma, Burlington, MA). Alternatively, exosomes were enriched from the supernatant obtained by centrifugation of serum as above and resolved by differential ultracentrifugation as previously described (51) with some modifications using a Beckman Coulter Optima LE-80k with a Beckman Coulter Type 50.2 Fixed Angle Rotor. Briefly, after the 12,000 × g spin of the clarified serum, the supernatant was centrifuged at 110,000 × g for 120 min. The pellet was resuspended in 2 mL of PBS containing 0.25 mM Trehalose (PBST) and centrifuged at 110, 000 × g for 70 min. The exosome pellet was resuspended in 200 μL of PBST. Purified exosomes from each protocol were used directly for RNA isolation, Western blotting or flash frozen and stored at −80°C for later analysis. A total of 5 groups per strain and 5 mice per group were utilized as biological replicates for the discovery as well as validation cohort.

### Total RNA Isolation

RNA was isolated using the miRNeasy Serum/Plasma Mini Kit (Qiagen, Hilden, Germany). The manufacturer's protocols were followed as written, except for the final collection step which was performed sequentially in two steps. First, 25 μL of nuclease-free water was added to the spin column for 10 min before elution of sample. Then, this step was repeated using 15 μL of nuclease-free water to increase the recovery yield. Combination of both eluates yielded around 30 μL of total RNA collected per pooled exosome sample from five mice. The amount and quality of RNA was analyzed using a Nanodrop to assess initial concentration, and TapeStation (Agilent) to assess sample quality utilizing RNA integrity number (RIN).

### Transmission Electron Microscopy

Exosomes stored at −80°C were thawed and fixed on 150 mesh copper carbon formvar grids (Electron Microscopy Sciences, Hatfield, PA). With high precision negative forceps (Electron Microscopy Sciences, Hatfield, PA), 10 μL of exosome samples were incubated with grids for 5 min. Excess liquid was absorbed using filter paper. The grid was incubated with 1% aqueous uranyl acetate (Electron Microscopy Sciences, Hatfield, PA) for 5 min. After rinsing with 10 μL ultrapure water, the grid was air dried for 30 min before storage or immediate viewing in a JEM1400 transmission electron microscope operating at 100 keV.

### Western Blotting

Equal volumes of exosome samples were heated for 5 min at 95°C under reducing conditions and resolved over 8–16% Novex WedgeWell Tris-Glycine Polyacrylamide Gels (ThermoFisher, Waltham, MA) for 90 min at 125 volts, under constant voltage. Proteins in gels were transferred to nitrocellulose membrane using an iBLOT 2 device and Invitrogen iBLOT 2 NC stacks (ThermoFisher, Waltham, MA). Membranes were rinsed in Phosphate Buffered Saline (PBS) and blocked in Rockland Blocking Buffer for Fluorescent Western Blotting (Pottstown, PA) for 1 h at room temperature. Membranes were incubated with rabbit primary polyclonal antibodies to TSG101 [Abcam—EPR7130(B), 1:250 dilution], and primary monoclonal antibody to Cathepsin L (Abcam—EPR8011, 1:500 dilution) overnight at 4°C. After six 5 min washes in 1x PBS, membranes were incubated in goat-anti rabbit IR800 secondary antibody for 1 h at RT and rinsed again with 1x PBS, 6 times for 5 min each before imaging on a LI-COR Odyssey Fluorescent Imager. Images were analyzed using ImageStudio v5.2.5.

### Particle Size Analysis

Size and concentration of exosomes was measured by Nanoparticle Tracking Analysis (NTA) using a ZetaView (Particle Metrix, Meerbusch, Germany). Some samples were also shipped to Alpha Nano Tech LLC (Chapel Hill, NC) for analysis by a ZetaView S/N 17-332 running the software ZetaView 8.04.02. After calibration with 100 nm standards (Applied Microspheres, The Netherlands), samples were diluted in varying amounts of PBS to reach the optimal concentration for analysis, then injected into the ZetaView cell for measurement. Eleven cell positions were sampled for two cycles each, with outliers automatically removed by the software. Measurements were taken at 22°C, using a sensitivity of 75, a frame rate of 30, and a shutter speed of 100. These measurements were analyzed using a minimum brightness of 20, a maximum size of 500 pixels, and a minimum size of 10 pixels. As it is the best determinant of particle size, the mode was selected as the main sizing parameter (52). Total particle count was calculated to account for varying resuspension volumes.

Particle size was also analyzed by Dynamic Light Scattering (DLS) using a Wyatt Dyna-Pro Plate reader II (Wyatt Technologies, Santa Barbara, CA). Briefly, 60 μL of exosome samples were run in triplicates at 25°C in a 384-well clear bottom plate (Greiner Bio One, Monroe, NC). The hydrodynamic radius of isolated exosomes was measured and presented as a normalized diameter. Data was analyzed using Dynamics V7 software (Wyatt, Santa Barbara, CA).

### Small RNA Deep Sequencing

Library preparation and sequencing on exosome fractions were performed by GeneWiz (South Plainfield, NJ). Total RNA containing the small RNA fraction was converted into cDNA using the Illumina TruSeq Small RNA library prep kit according to the manufacturer's instructions. Briefly, 3' adapter “RA3” and 5' adapter “RA5,” were ligated to total RNA which was then reverse transcribed. Adapter ligated cDNA library was enriched by PCR using primers that selectively anneal to the adapter sequence and then purified by gel electrophoresis. Quality of the cDNA library was assessed using a DNA chip on bioanalyzer. Barcodes were added to each sample and all 10 samples were sequenced on a single lane of an Illumina HiSeq system set to a 2 × 150 bp configuration. The output generated a total of ~414 million reads which were then demultiplexed with the added barcode separating the files according to the samples into FASTQ files.

### Bioinformatics

The raw FASTQ files obtained from Genewiz were assessed for their quality using FastQC v0.11.9. Adapter trimming was performed using Cutadapt v2.8 (https://github.com/marcelm/cutadapt). High quality reads of minimum length 15 nucleotides (nt) were mapped to whole genome (mm10 assembly GRCm38) using Bowtie v1.2.3 (53) and annotated using featureCounts v2.0.0 (54), using GENCODE (Release M24, GRCm38.p6) comprehensive gene annotation GTF file (PRI) to obtain distribution of reads over genome and raw counts for various non-coding RNA such as pre-miRNA, scRNA, scaRNA, snRNA, tRNA, rRNA, snoRNA, and lncRNA. Reads were then mapped to small RNA transcriptomes (miRbase v22, piRdbv2.0). The output files in the SAM file format were sorted to mapped reads that had an alignment CIGAR string of 18M or higher. Sam2counts, a python program (https://github.com/vsbuffalo/sam2counts), was used to acquire counts of reads aligned to transcriptomes (55).

Raw reads were also aligned to the piRNA and miRNA transcriptomes using an in-house aligner “miRGrep” (https://github.com/singhkakan/miRGrep) that applies brute force to count the number of reads containing a given miRNA or piRNA sequence. As miRNA are 19–26 bp long and piRNA are 24–30 bp long, the entirety of their sequence is read during sequencing. As a result, the reads (75 to 150 bp) are longer than the miRNA or piRNA of interest and contain their complete sequence. miRGrep yielded a final count table which was assembled in RStudio using the dplyr package for further processing. After the miRNA or piRNA counts table was generated, differential gene expression analysis was conducted using three statistical R packages DESEq2 (56), EdgeR (57), and LimmaVoom (58) in RStudio. Statistical significance was determined by adjusted *p* < 0.05 by DESEq2 or Limma or False Discovery Rate (FDR) <0.1 by EdgeR. We have included miRNAs considered significant by at least 1 statistical package for downstream analyses. The experimental procedures and analysis pipeline are detailed in Supplementary Figure 1.

### miRNA Validation Assays

In a separate validation cohort of 5 groups with five mice per group, we isolated serum exosomal RNA. The differential expression of miRNAs of interest was validated by qRT-PCR using individual Taqman Advanced miRNA Assays (Applied Biosystems). Briefly, poly-A tailing and adapter ligation was performed on 2 μL of total RNA isolated from serum exosomes of the validation cohort using the Taqman Advanced cDNA synthesis kit. Following this cDNA synthesis, miRNA amplification was conducted using the same kit. The amplified cDNA was diluted 1:10 and set up in triplicate qRT-PCRs with 1 μL of specific Taqman Advanced miRNA primer and run on a Quant-Studio Flex 6 (Applied Biosystems, Foster City, CA), using the assay's recommended cycling conditions. Results were analyzed by the ΔΔCt method with BALB/c serum exosomal small RNA as reference and miR-16-5p as housekeeping miRNA. 16-5p is identified in the literature as a suitable housekeeping miRNA (59–62) and was unchanged between serum exosomes in BALB/c and NOD mice in the sequencing data obtained in this study (*p* = 0.995, DESeq2).

### Pathway and Functional Enrichment Analysis

Pathway analyses were conducted using miTALOS v2.0 (63) using StarBase2, a database of experimentally validated miRNA targets. Pathway data were extracted from KEGG, Reactome, and WikiPathways by miTALOS. Pathways with a corrected *p* < 0.05 and Enrichment score > 1 are expected to contain over-represented miRNA targets. Functional enrichment analysis was done using the custom heatmap calculator of miRPathDB v2.0 (64) using GeneOntology (Biological Enrichment) with at least two miRNA per pathway and two pathways per miRNA. The settings were chosen such that signaling pathways in which at least 2 of our miRNAs of interest have an mRNA target, would be identified. Additionally, the program was directed to identify at least two pathways targeted by each miRNA. With these constraints we may identify pathways that have a high probability of being dysregulated with the aberrant expression of the miRNA of interest. Pathway analysis was also conducted using Ingenuity Pathway Analysis (IPA) to visualize the interaction of miRNA “hits” with their targets in the signaling pathways relevant to autoimmunity, which were also identified by miTALOS and miRPathDB.

## Results

### Characterization of Exosomes From Mouse Serum

**Figure 1:**
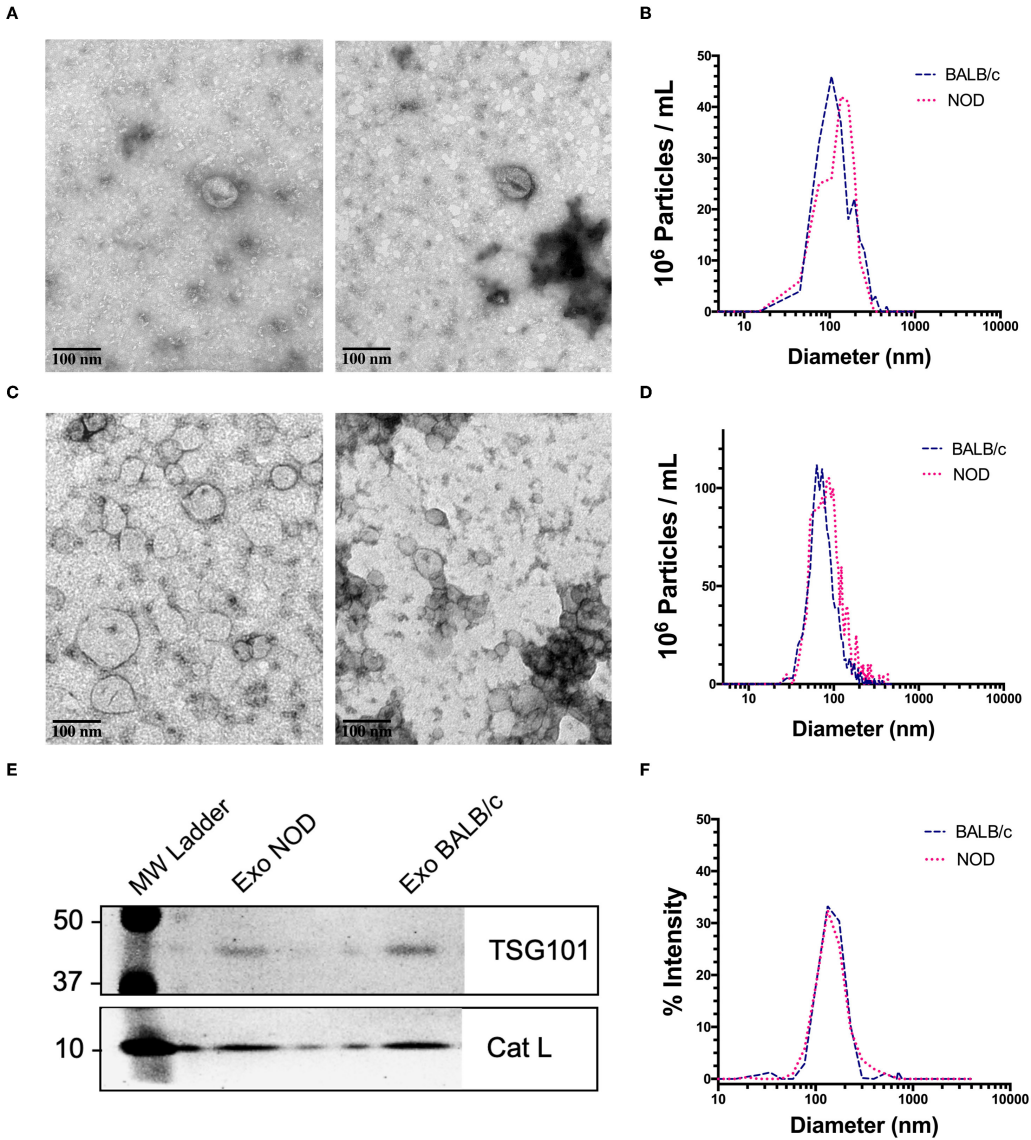
Characterization of mouse serum-derived exosomes by differential Ultracentrifugation (UC) and Sice Exclusion Chromatography (SEC). **(A)** Transmission Electron Microscopy (TEM) images of exosomes isolated from NOD (left) and BALB/c (right) mouse serum using differential UC. The cup and saucer shaped morphology typical of exosomes is visible. **(B)** NTA showed median diameters of 136 and 131 nm for serum exosomes from NOD and BALB/c mice, respectively. The graph is representative of four separate experiments per mouse strain. No significant differences in exosome median diameter were observed between the two strains. **(C)** TEM images of SEC exosomes isolated from NOD (left) and BALB/c (right) mouse serum. **(D)** NTA of SEC exosomes. **(E)** Western blotting of NOD and BALB/c serum exosomes isolated by SEC show enrichment of TSG101 and Cathepsin L. **(F)** Exosome particle size of SEC exosomes analyzed by DLS showed a median diameter of 122 nm from both strains. The graph is representative of three separate experiments.

We chose exosomes as the principal source of serum miRNAs based on findings that miRNAs are concentrated in these organelles in extracellular biofluids. Exosomes were isolated by differential ultracentrifugation (UC) for discovery experiments and by size exclusion chromatography (SEC) for validation experiments. Transmission electron microscopy (TEM) images showed ~100 nm sized vesicles with the characteristic cup shaped morphology typical of exosomes (65) for both UC (Figure 1A) and SEC exosomes (Figure 1C). UC exosomes had a median diameter of 136 nm from male NOD mouse serum and 131 nm for BALB/c mouse serum, while SEC exosomes had a median diameter of 122 nm for NOD and BALB/c mouse serum (Figures 1B,D). There was no significant difference in the sizes of the exosomes isolated by the two methods, although exosomes isolated by UC were slightly larger in accord with previous reports (66). SEC has been reported to give a better yield than UC (67), consistent with our findings. There were no significant differences in the size or concentration of exosomes between the two strains of mice. Western blotting also showed the presence of the universal exosome membrane protein marker, TSG101, in exosomes isolated from both strains (Figure 1E). Various cathepsins (S, D, K and L) have been found in exosomes derived from plasma (68), macrophages (69), and microglia (70). Interestingly, we found enrichment of cathepsin L in exosomes isolated from both strains (Figure 1E). DLS found that SEC exosomes ranged from 60 to 130 nm with a mean diameter of 122 nm (Figure 1F). There was no strain specific difference in size, concentration or marker expression between exosomes isolated from the two strains. For study design, UC exosomes were used for miRNA “hit” identification and SEC exosomes were used for “hit” validation.

### Mouse Serum Exosomes Contain Several Small RNA Biotypes

**Figure 2:**
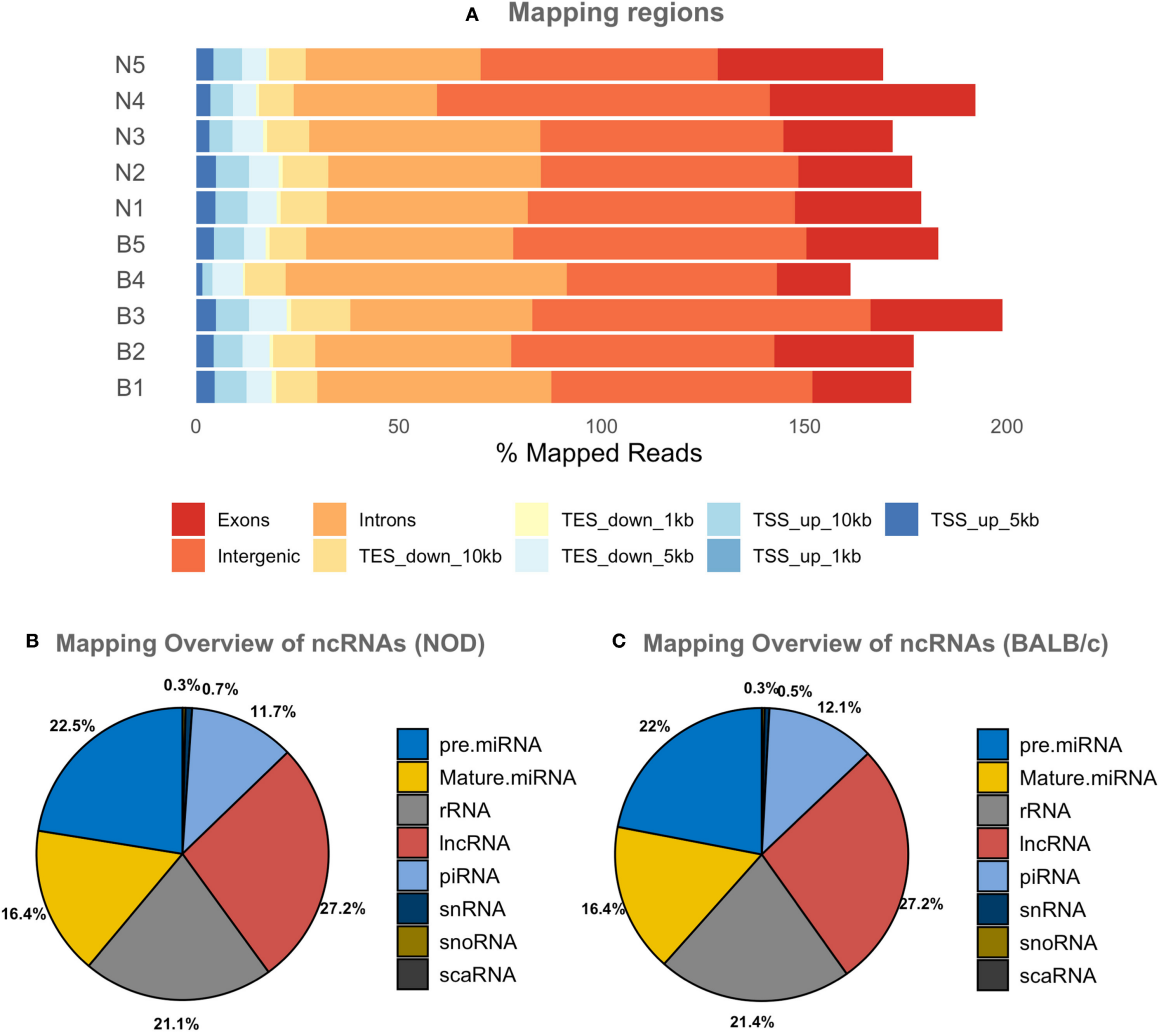
Graphical presentations of the distribution of reads from NGS sequencing of RNAs. **(A)** Shows the regions of the mouse genome that reads of all samples were aligned to. The mapping percentage is >100 as multi-mapping reads were also counted. No significant sample to sample variation was observed. **(B,C)** Show the distribution of reads aligning to various ncRNAs in NOD & BALB/c serum UC exosomes. Results were obtained from *n* = 5 UC serum exosome samples per mouse strain, with each sample comprising pooled serum exosomes from five mice. TSS, Transcription Start Site; TES, Transcription End Site; kb, kilobases; pre miRNA, precursor microRNA; rRNA, ribosomal RNA; lnc RNA, long non-coding RNA; snRNA, small nuclear RNA; snoRNA, small nucleolar RNA; scaRNA, small cajal body specific RNA; mature miRNA, mature microRNA; piRNA, piwi interacting RNA.

Since most extracellular ncRNA are associated with extracellular vesicles (71), UC serum exosomes were used as a source for small RNAs from NOD and BALB/c mice. Establishment of autoimmune dacryoadenitis, the most notable characteristic of SS in these mice, was confirmed in each NOD cohort relative to BALB/c by H & E staining of lacrimal gland sections and quantitation of lymphocytic infiltrates (Supplementary Figure 2). While we did not observe any lymphocytes infiltrating LG in the BALB/c, NOD mice had infiltration between 8 and 15% (*p* < 0.0001, one-way ANOVA). Using next-generation sequencing (NGS), we profiled small RNA in serum exosomes from each mouse strain to identify both novel and differentially expressed miRNA. A total of 417 million raw reads were generated which were mapped to the mouse genome and various small ncRNA transcriptomes. Of the reads that mapped to the mouse genome, roughly two-thirds of the reads mapped to intergenic or intronic regions while a third of the reads mapped to exons. Less than 10% of the reads mapped to transcription start or end sites (Figure 2A). Read distribution was fairly uniform across samples with no strain specific difference. More than 50% of mapped reads were comprised of miRNA (both mature and precursor) and piRNA. Nearly 27% of reads mapped to lncRNA, and 20% to rRNA, whereas <1% mapped to small nucleolar RNA (snoRNA) (Figures 2B,C) in serum exosomes of both NODs and BALB/c mice. Reads that mapped to miRNA, piRNA, lncRNA, snRNA, snoRNA, scaRNA and rRNA are provided in Supplementary Table 1. We did not observe any strain-specific differences in proportion of RNA sub-types within the small ncRNA libraries of NOD and BALB/c serum exosomes (*p* = 0.925, ordinary two-way ANOVA).

piRNA are involved in gene silencing of transposons by forming complexes with argonaute proteins (72), and appear to provide RNA-mediated adaptive immunity against transposons (73). In our analysis we found that 208 piRNA were expressed in NOD mouse UC exosome samples and 238 in BALB/c mouse samples. Of these, 171 were found in both but 37 were unique to NOD mouse samples, and 67 unique to BALB/c mouse samples (Supplementary Figure 3B). The 10 most highly expressed piRNA did not appear to be dysregulated in the NOD strain (Supplementary Figure 3A). There were no significant differences in the total number of distinct piRNA or the total number of reads aligning to piRdb between the two strains (Supplementary Figure 3C). We found 13 piRNA to be upregulated in NOD mouse samples with a log_2_ fold change >3, while 15 were downregulated with a log_2_ fold change < −3. Of these, only mmu-piR-58696 was significantly upregulated in the NODs as determined by LimmaVoom (p_adj_ = 0.047) (Supplementary Figure 3D).

### NOD Serum Exosomes Contain a Subset of Dysregulated miRNA

FASTQ reads were preprocessed using FASTqc which identified the presence of adapter in ~90% of the reads. Cutadapt was used to remove adapter sequences and exclude reads of quality <20 and length <15. Pre-processed trimmed reads were then mapped to the miRNA transcriptome from miRbase v22.0 using Bowtie v1.2.3, as well as our in-house aligner miRGrep, which utilizes brute force and was written specifically for the alignment of RNA < 30 nt in length such as miRNA and piRNA. With Bowtie, we identified 550 distinct miRNAs in NODs and 255 in BALB/c. Using miRGrep, we identified 251 miRNAs in the NODs and 242 miRNAs in BALB/c. This is to be expected because miRGrep uses brute-force to align reads to miRNA, with no mismatch allowed in alignment and therefore, the miRNA identified by miRGrep are a subset of those identified by Bowtie (74). Of these, read counts for 38 miRNAs were found only in NOD serum exosomes while 29 miRNAs were found only in BALB/c serum exosomes (Supplementary Figure 4A) in at least 3 out of 5 sample groups per strain. The top 20 expressed miRNA in NOD serum exosomes were overrepresented to the same extent in BALB/c (Supplementary Figure 4B) with no discernible strain specific differences. Of these miRNAs, miR-191-5p, miR-92a-3p, miR-22-3p, miR-16-5p, let-7f-5p, let-7i-5p, miR-26a-5p, miR-30e-5p, miR-186-5p, miR-30d-5p, miR-451a, miR-181a-5p, miR-148a-3p, miR-423-5p, let-7a-5p, and miR-25-3p have been previously reported to be abundant in serum exosomes (75). miR-486-5p (not shown) was the top over-represented miRNA as reported previously (75). It is possible that these miRNAs serve important regulatory functions that are evolutionarily conserved.

**Figure 3:**
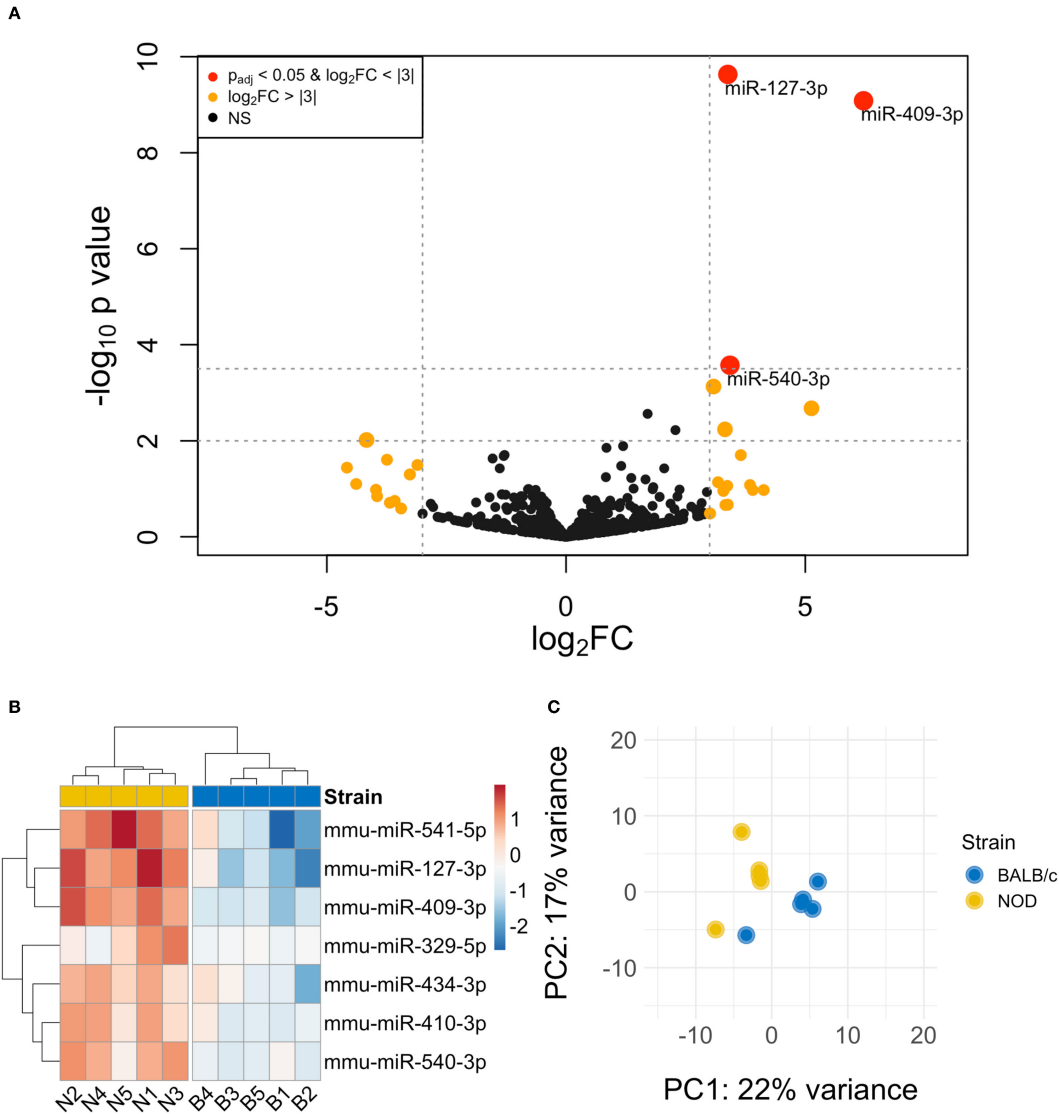
Differential miRNA expression analysis. **(A)** The volcano plot highlights miRNAs that were identified in exosomes isolated by differential ultracentrifugation as upregulated or downregulated by more than 3-fold, shown in orange. Upregulated miRNAs that are statistically significantly different are shown in red. **(B)** Unsupervised hierarchically clustered heatmaps of the most differentially expressed miRNA (with highly expressed miRNAs in red and minimally expressed miRNAs in blue) in the 10 samples. Samples are labeled either N for NOD or B for BALB/c. Individual samples for the top differentially expressed miRNA cluster by strain. **(C)** PCA plot of all miRNAs shows that 22% of the variance was accounted for by strain.

**Figure 4:**
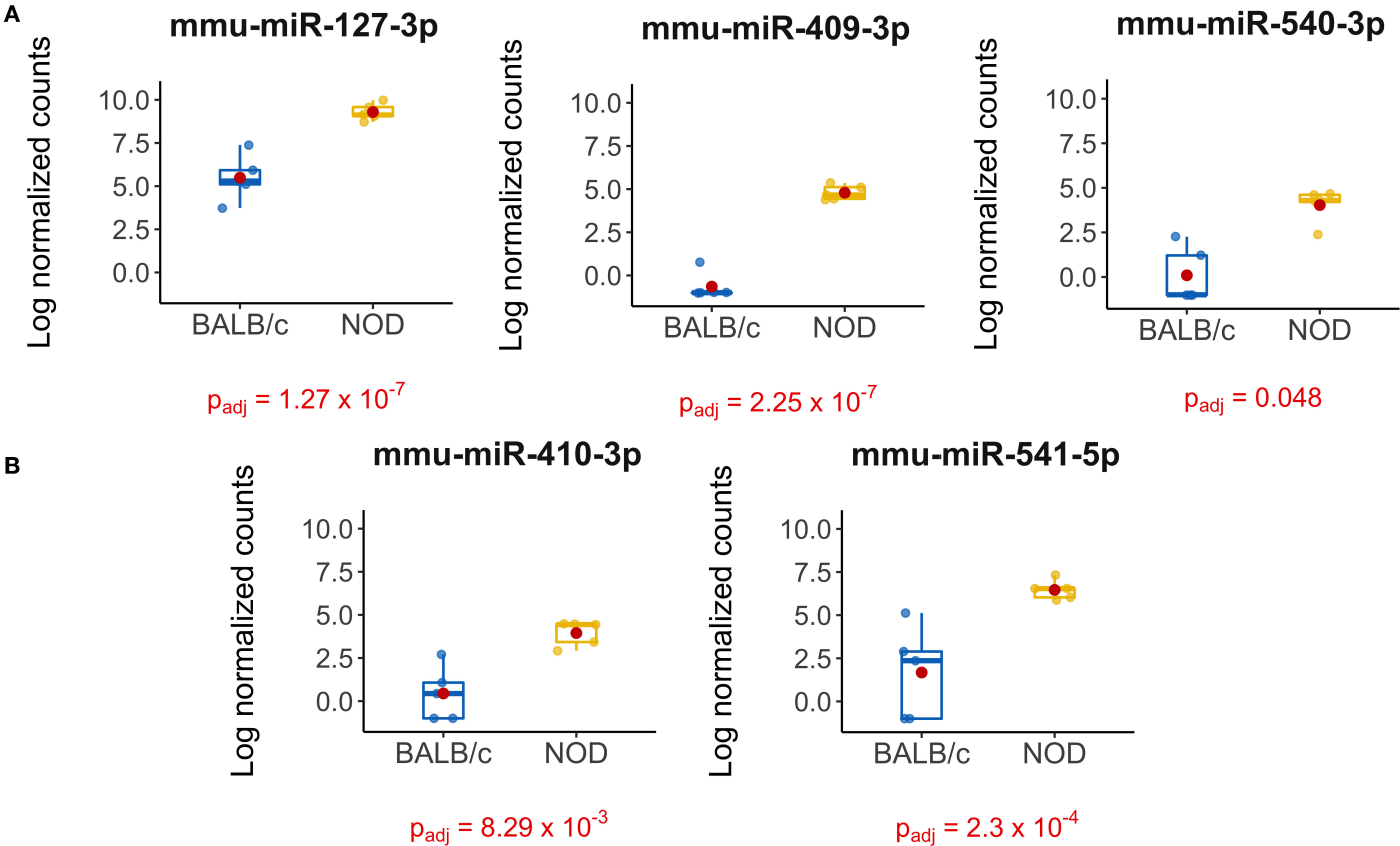
Boxplots of differentially expressed miRNAs in exosomes isolated from 14-week male BALB/c and NOD mouse serum. Reads were aligned with Bowtie and an in-house brute force-based aligner, and differential gene expression analysis was conducted using DESEq2, EdgeR, and LimmaVoom in RStudio. **(A)** Both DESeq2 and LimmaVoom identified miR-127-3p, miR-409-3p, and miR-540-3p as “hits” with p_adj_ < 0.05. **(B)** Additionally, LimmaVoom identified miR-410-3p and miR-541-5p to be dysregulated with p_adj_ < 0.05. miR-329-5p failed to reach statistical significance due to one outlier but exhibited the highest fold change with Log_2_FC > 5 in EdgeR and DESEq2. Results were obtained from data sets generated from small RNA sequencing of 5 sets of RNA from exosomes from each mouse cohort, each isolated from purified pooled serum exosomes from five groups of age-, gender-, and strain-matched mice, with 5 mice per group.

The volcano plot in Figure 3A shows the level of differential expression for all expressed miRNA identified in NOD mouse serum. miRNA that had an adjusted *p* > 0.05 and log_2_ fold change <3 or >-3 were not considered significant. DESeq2 and Limma determine significance when an adjusted *p* < 0.05 is reached whereas EdgeR considers a hit significant only when the FDR is <0.1 in RStudio (Table 1). We have reported and assessed miRNA that met our significance criteria by at least one of the statistical packages. Unsupervised hierarchical clustering analysis of top hits using the Euclidean method clustered NOD mouse miRNA samples in the same group, separate from BALB/c samples, as shown by the top tree (Figure 3B), suggesting that the differential expression observed may be attributed to dysregulation in the NOD strain. We performed an additional unsupervised technique to visualize the variability between the two groups. Principal component analysis (PCA) of the 10 samples with ~500 expressed miRNAs revealed that 22% of the variance could be explained by the differences in strain (Figure 3C). All three packages determined that miR-127-3p, miR-409-3p, and miR-540-3p were significantly overexpressed in NOD mouse (Figure 4A). Limma identified miR-410-3p and miR-541-5p to be overexpressed in NOD serum exosomes (Figure 4B).

**Table 1:** Summary of the 7 most differentially-expressed miRNAs detected in serum UC exosomes from 14-week male BALB/c and NOD mice using three statistical packages in RStudio.

	**DESeq2**		**Limma**		**EdgeR**	
**miRNA**	**Log_**2**_ FC**	**p_**adj**_**	**Log_**2**_ FC**	**p_**adj**_**	**Log_**2**_ FC**	***p*-val**
miR-127-3p	3.38	**1.27 × 10**^**−7**^	3.77	**6.08 × 10**^**−6**^	3.40	**2.14 × 10**^**−4**^
miR-409-3p	6.22	**2.25 × 10**^**−7**^	5.41	**4.38 × 10**^**−6**^	6.16	**5.06 × 10**^**−5**^
miR-540-3p	3.42	**0.0485**	3.67	**5.0 × 10**^**−3**^	3.37	**1.39 × 10**^**−3**^
miR-410-3p	3.08	0.101	3.49	**8.29 × 10**^**−3**^	3.02	2.49 × 10^−3^
miR-541-5p	3.32	0.406	5.34	**2.3 × 10**^**−4**^	3.24	8.88 × 10^−3^
miR-329-5p	5.13	0.229	–	–	6.23	1.2 × 10^−2^
miR-30d-3p	−4.17	0.580	−2.67	0.196	−4.4	5.79 × 10^−3^

*Values reaching statistical significance at p_adj_ < 0.05 are bolded, for DESeq2 and Limma Significance for EdgeR is determined by False Discovery Rate < 0.10*.

Furthermore, we found an additional 19 miRNAs that displayed an at least 3-fold higher expression in NOD serum exosomes than in Balb/c and 11 miRNAs that were under-expressed by at least 3-fold in NOD mice (Supplementary Figure 4C). Despite a meaningful fold change these did not reach statistical significance due to an outlier. Of these, miR-329-5p was found to be over 5-fold overexpressed in NOD serum exosomes (DESeq2, EdgeR). As NOD mice, even when age matched, show a variation in disease progression, it is possible that the outlier group may have progressed further in disease than other groups and vice versa. Thus, we included miR-329-5p in our downstream analyses as its differential expression may have biologically significance.

### Validation of miRNA Differential Expression

**Figure 5:**
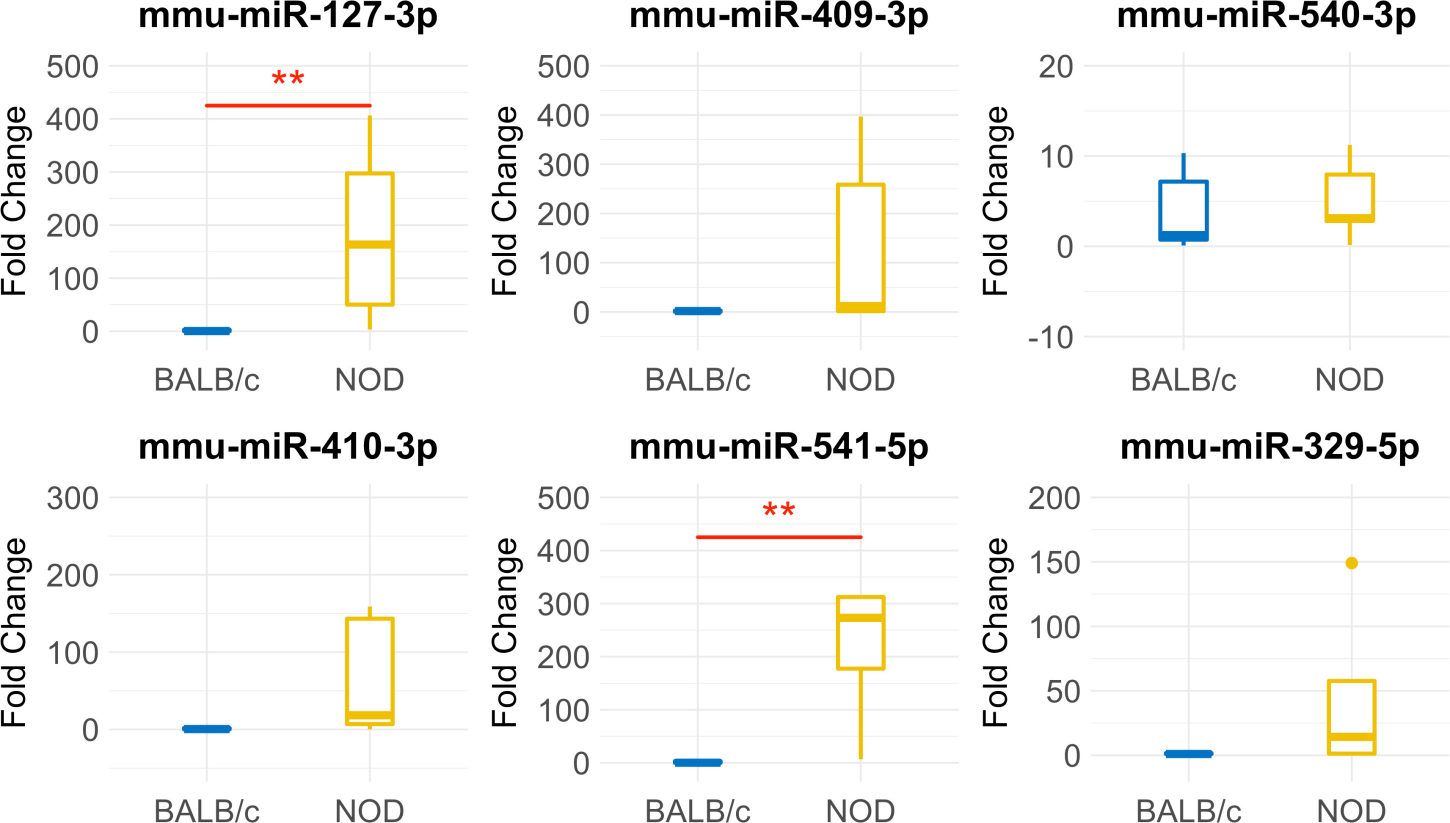
Validation of miRNA ‘hits’ in Size Exclusion Chromatography (SEC)-purified serum exosomes from 14-week male BALB/c and NOD mice. Fold-change data represent mean ± SD normalized to miR-16-5p using the ΔΔCt method. Statistical significance was determined by the Mann-Whitney U-test; **p < 0.01. Results were obtained from data sets generated from qRT-PCR from RNA isolated from SEC-purified pooled serum exosomes from five groups of mice per strain, with 5 mice per group. All mice were age matched.

To validate our deep sequencing findings of known miRNAs that were differentially expressed in NOD vs. BALB/c mouse serum exosomes, we purified serum exosomes from independent cohorts of mice, 5 samples per strain with each sample comprised of serum exosomes from each of five mice. For this round of exosome isolation, we used the SEC method (Figures 1C–F) which yielded exosomes of greater particle homogeneity. qRT-PCR was performed using Advanced Taqman miRNA Assays. In agreement with the sequencing data, miRNA miR-127-3p, miR-409-3p, miR-540-3p, miR-410-3p, miR-541-5p, and miR-329-5p were expressed at a higher level in NOD mouse serum exosomes (Figure 5). Five out of the six miRNAs were more than 25-fold over-expressed in the NODs on average. Of these, over-expression of mmu-miR-127-3p and mmu-miR-541-5p were found to be statistically significant (*p* < 0.01, Mann-Whitney U-test). miR-410-3p and miR-329-5p were upregulated in 4/5 groups whereas miR-409-3p was upregulated in 3/5 groups.

### Pathway Analysis Identifies Several Pathways Involved in Lymphocyte Activation

To understand the signaling pathways the “hit” miRNA may be involved in, we used three methods (miTALOS, miRPathDB, and IPA) which utilize a range of databases of predicted and experimentally validated mRNA-miRNA interactions (such as Tarbase, TargetScan, miRanda, Starbase2) and databases of known pathways (KEGG, Reactome, WikiPathways) in their pathway analysis algorithm. These databases do not have information on all known miRNA for every species, and so we used multiple tools in our pathway analysis to be as comprehensive as possible. Functional over-representation analysis using Gene Ontology (Biological Functions) in miTALOS allows miRNA target prediction using the latest versions of TargetScan (6.2) and miRanda. Additionally, a database of experimentally validated targets—StarBase2- was also implemented in the pathway analysis and is available for both human and murine microRNAs. StarBase2 catalogs 3 miRNA hits identified in this study –mmu-miR-127-3p, mmu-miR-409-3p, mmu-miR-410-3p. As these have identical sequences for human and mice, pathway analysis for both species (Supplementary Figures 5A,B) was conducted using these three hits. Databases in miRPathDB catalog mmu-miR-541-5p in addition to the three miRNAs above. Using miRPathDB, both KEGG (not shown) and WikiPathways identified B cell receptor signaling (Supplementary Figure 5C) while Gene ontology—Biological functions identified lymphocyte activation (not shown). Other pathways identified are associated with cellular proliferation, pluripotency, apoptosis, and p53 signaling, all pathways that may be relevant to cancer. IPA identified several pathways involved in immune regulation (Supplementary Figure 5D). Pathways common to all three analyses include B-Cell Receptor (miR-targeted genes in lymphocyte in miTALOS), TGF-beta and IL-6 Signaling.

## Discussion

Here we report that an unbiased screen of exosomal serum miRNA in a murine model of early-intermediate stage autoimmune dacryoadenitis and SS, identified the significant upregulation of multiple miRNAs. qPCR analysis of serum exosomes from a separate set of disease-model vs. healthy control mice validated two significant hits, miR-127-3p and miR-541-3p, while confirming marked elevation with some variance across animal groups for three additional miRNAs, miR-409-3p, miR-410-3p, and miR-329-5p. SS is a complicated autoimmune disease, with treatment hindered by a poorly understood pathogenesis and a lack of reliable diagnostics. Given that miRNAs are master regulators of gene expression with their dysregulation implicated in many diseases (76), identification of this group of dysregulated miRNAs in the male NOD mice at the initial stages of autoimmune dacryoadenitis and development of other indicators of established systemic disease in SS may be useful in establishment of future diagnostic biomarkers.

mmu-miR-127-3p, a significant and validated hit, has been shown to be necessary for the self-renewal and differentiation of hematopoietic stem cells (HSC) in a mouse model of HSC self-renewal defect (77). It has also been proposed to be a regulator of senescence as a tumor suppressor by directly targeting BCL6 (78, 79), a known protooncogene in human cell lines. As BCL6 is a transcriptional repressor and inhibits the production of IL10, its downregulation by miR-127-3p can lead to an increase in IL-10 (80). Increased levels of IL-10 are well-documented in SS patients (81, 82) and are also reported in the NOD mouse lacrimal gland in association with development of autoimmune dacryoadenitis (15). Our pathway analysis shows that mmu-miR-127-3p, is involved in the regulation of TGF-beta and B-Cell receptor Signaling (Supplementary Figure 5C) through its targeting of several MAP kinases and BCL6 (Supplementary Figure 5D). Appropriate regulation of BCL6 is also necessary for the development of germinal center B cell and follicular helper T cells (83). Upregulated levels of hsa-miR-127-3p are reported in testicular and nodal diffused large B-cell lymphoma, with an inverse correlation to BCL6 levels (84). Thus, regulated levels of miR-127-3p are necessary for appropriate control of lymphoproliferation and B cell homeostasis and elevated levels may be indicative of immune dysfunction/autoimmunity. This finding in the NOD mice is of particular relevance because a subset of SS patients develop B cell lymphoma (5) and there is great interest in biomarkers that may distinguish these patients from others with SS so that earlier interventions may be applied to suppress development of B cell lymphoma. It will be of great interest to study the potential dysregulation of miR-127-3p longitudinally in SS patients to explore its relationship to this debilitating and most destructive manifestation of SS.

mmu-miR-541-5p, a second significant and validated hit, may work in concert with mmu-miR-127-3p. In a mouse model of multiple sclerosis, miR-541-5p and miR-127-3p were upregulated in lymph nodes indicating that they may be involved in pathogenic neuro-inflammation (85). Interestingly, knockout of TNF-α in a mouse model led to downregulation of both miR-541-5p and miR-127-3p in epidermal skin, hinting at a close involvement of these miRNAs with pro-inflammatory cytokines (86).

Another hit, miR-409-3p, is broadly implicated in autoimmune disease in animal models and patients with chronic fatigue syndrome/myalgic encephalomyelitis (87), multiple sclerosis (88) and systemic lupus erythematosus (89). Our pathway analysis using miRPathDB and IPA indicates miR-409-3p's involvement in the B Cell Receptor, STAT3 and IL-6 signaling pathways (Supplementary Figures 5C,D). Studies have found that in mice with experimental autoimmune encephalomyelitis (EAE, a murine model of multiple sclerosis), mmu-miR-409-3p targets suppressor of cytokine signaling protein 3 (SOCS3). Upregulation of mmu-miR-409-3p in astrocytes of EAE mice silences SOCS3, leading to an increase in phosphorylation of STAT3 and increased production of inflammatory cytokines such as IL-1β, CXCL10, IL-6, MC-P1 (90). Another study found that in a co-culture of NOD mice salivary gland acinar cells (SGAC) and B-lymphocyte (an *in-vitro* model of salivary gland disease in SS), there was a significant increase in production of cytokines IL-6 and IL-1β by B-lymphocytes and increased phosphorylation of STAT3 in SGAC (91). IL-1β is upregulated in diseased NOD mouse LG, while its injection into murine LG further impairs tear production (92). Secretion of these cytokines by circulating lymphocytes is also increased in SS patients (93). Thus, mmu-miR-409-3p may be pro-inflammatory in nature and its upregulation may increase cytokine production via the SOCS3/STAT3 signaling pathway.

According to TargetScan, mammals including mice and humans have an 8-mer conserved site on the Stat3 gene for miR-410 (30). hsa-miR-410-3p appears to directly target STAT3, leading to a reduction of IL-10 in T cells of patients with systemic lupus erythematosus (94), and was also shown to be elevated in the plasma of these patients (95). Increased expression of miR-410-3p was also observed in males with relapsing remitting multiple sclerosis, which is characterized by cycling of autoimmune inflammatory status (96). Expression of hsa-miR-410-3p was decreased in the synovial fluid and synoviocytes of rheumatoid arthritis (RA) patients. On the other hand, in an *in-vitro* model of RA, overexpression of hsa-miR-410-3p decreased the pro-inflammatory cytokines, TNF-α, IL-6, IL-1β, and MMP-9 (97) and was anti-proliferative and apoptotic in nature through targeting of transcription factor YY1 (98). Based on these results, upregulated miR-410-3p may have an immune-protective effect. If validated, use of this miRNA may have value as a potential therapeutic.

Of interest, five of the identified miRNAs (miR-127-3p, miR-329-5p, miR-409-3p, miR-410-3p and miR-541-5p) are encoded on the highly evolutionarily conserved Dlk1-Gtl2 locus on the maternally inherited allele of mouse chromosome 12, which is analogous to the locus Dlk1-Dio3 on the maternally inherited allele of human chromosome 14. The miRNAs from this locus seem to regulate ground state pluripotency in embryonic stem cells (99). Genes from this imprinted locus play a critical role in embryonic and fetal development and appear to be dysregulated in several diseases, including blood cancers such as lymphoma, acute myeloid and acute promyelocytic leukemia, as well as autoimmune diseases such as lupus nephritis (100) and multiple sclerosis (96). It is also interesting that the sequences of mature miRNA 127-3p, 409-3p, and 410-3p are identical in human and mouse. Sequences of miRNA-541-5p and miRNA-329-5p vary only by 3 nucleotides between human and mouse. This further highlights the applicability of miRNA-based biomarkers arising from murine model in this study to humans.

Studies of extracellular vesicles rely on particle size analysis which cannot discriminate between functional vesicles and lipid droplets of similar size, and the presence of these can be a confounding factor in our studies. Future studies will aim to validate these results utilizing different methods of exosome isolation. Although our choice of serum over plasma and the use of serum-separator tubes was aimed at reducing hemolysis, it may also be a confounding factor in our study. While miRNA stability studies in plasma have shown that levels of miR-127-3p are not altered by hemolysis (87), strengthening its potential as a biomarker candidate, similar investigations are needed for the other miRNAs identified in this study.

While we have identified the potential role of some miRNA in inflammatory pathways and SS, further study is required to better understand the relationships of these miRNA as well as the temporal changes that occur with disease progression. The panel of miRNA identified in this study reflect early changes in SS progression, while different patterns of dysregulation may be observed longitudinally as disease advances and/or as it impacts different organs. Since the secretory contents from both lacrimal and salivary glands are also influenced by the autoimmune inflammation characteristic of SS and have been used as sources of biomarkers to reflect both systemic and local inflammation, evaluating these biofluids in later studies as additional sources of potential biomarkers will be of importance. Future studies will also focus on assessing the utility of this panel of miRNA in identifying SS patients as well as exploring the utility of these miRNA in longitudinal disease progression in combination with existing blood-based biomarkers such as anti-La and anti-Ro antibodies and others.

## Data Availability Statement

The original contributions presented in the study are publicly available. The datasets can be found in the Sequence Research Archive under ID PRJNA622527 and are available for download here https://www.ncbi.nlm.nih.gov/bioproject/622527.

## Ethics Statement

The animal study was reviewed and approved by University of Southern California, Institutional Animal Care and Use Committee (IACUC).

## Author Contributions

SK performed the bioinformatics, statistical analysis of next generation sequencing data, isolated and characterized mouse serum exosomes from the validation mouse cohort, isolated exosomal RNA, performed qPCR validation, and prepared the manuscript and supplementary files. SJ and BC isolated and characterized mice serum exosomes and exosomal RNA from the test cohort. SJ assisted with blood collection and performed qPCR validation of miRNA in the validation cohort. BC and SK optimized serum exosome isolation protocols. ME contributed to writing the manuscript and characterization of serum exosomes. DC contributed to data analysis and revised final manuscript. CO and SH-A secured research funding and contributed to experimental design and writing the manuscript. SH-A revised the final manuscript. All authors contributed to the article and approved the submitted version.

## Conflict of Interest

The authors declare that the research was conducted in the absence of any commercial or financial relationships that could be construed as a potential conflict of interest.
