# Supplementary material for: Small RNA Deep Sequencing Identifies a Unique miRNA Signature Released in Serum Exosomes in a Mouse Model of Sjögren's Syndrome
Source: Front Immunol. 2020 Jul 17;11:1475. doi: 10.3389/fimmu.2020.01475 (PMC7396589; doi:10.3389/fimmu.2020.01475)
Supplement: Supplementary file 1 [file Data_Sheet_1.PDF]

Supplementary Material

Small RNA deep sequencing identifies a unique miRNA signature released in serum exosomes in a mouse model of Sjögren's Syndrome

Shruti Singh Kakan[1], Srikanth R. Janga[2], Benjamin Cooperman[1], David W Craig[3], Maria C. Edman[2], Curtis T. Okamoto[1], and Sarah F. Hamm-Alvarez[1,2*]

[1]Department of Pharmacology and Pharmaceutical Sciences[1], School of Pharmacy, University of Southern California, Los Angeles, CA, USA

[2]Roski Eye Institute, Department of Ophthalmology[2], Keck School of Medicine, University of Southern California, Los Angeles, CA, USA

[3]Department of Translational Genomics[3], Keck School of Medicine, University of Southern California, Los Angeles, CA, USA

* Correspondence:
Sarah F. Hamm-Alvarez
shalvar@usc.edu

Keywords: Sjgren's Syndrome; diagnostic miRNA biomarkers; extracellular vesicles; small-RNA sequencing; microRNA; piwi-RNA

1 Supplementary Figures and Tables

1.1 Supplementary Figures

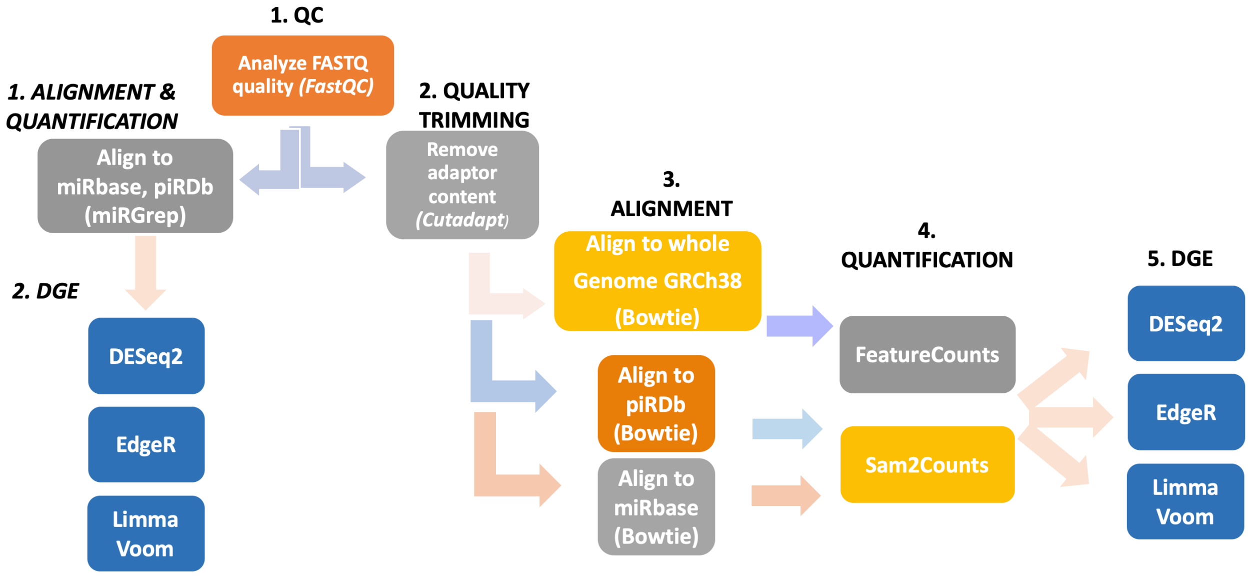

Supplementary Figure 1. Schematic depicting the bioinformatics analysis pipelines. Mouse serum was collected by cardiac puncture and exosomes were isolated by differential ultracentrifugation (UC). Exosomal RNA was isolated and assessed for quality. After library preparation, Illumina HiSeq was used for sequencing 150 bp paired end reads. Data analysis was done using Bowtie and an in-house aligner miRGrep. (1) Quality of reads was assessed using the program FastQC (v0.11.9). The QC found that the samples had adapter content and required trimming. (2) Adapter content and reads with poor quality scores were removed using Cutadapt (v2.8). (3) Trimmed reads were aligned to whole mouse genome (GRCm38.p6) to identify the regions of the genome the reads map to (exons vs introns vs intergenic) using Bowtie (v1.2.3). (4) Counts for various non-coding, RNA (including pre-miRNA snoRNA, scaRNA, scRNA, snRNA, rRNA, tRNA) were obtained using featureCounts (v2.0.0). Reads were aligned to miRBase v22 using Bowtie and count table was generated using a python program sam2counts. (5) Differential gene expression analysis was conducted in RStudio using DESeq2, EdgeR and Limma.

Alternately, *(1)* alignment and quantification of reads to miRBase v22 and piRDb v2 was done using an in-house aligner miRGrep that employs brute force as its alignment algorithm and generates a final count table. *(2)* Differential Gene expression analysis was done similarly as before in RStudio using three statistical packages.

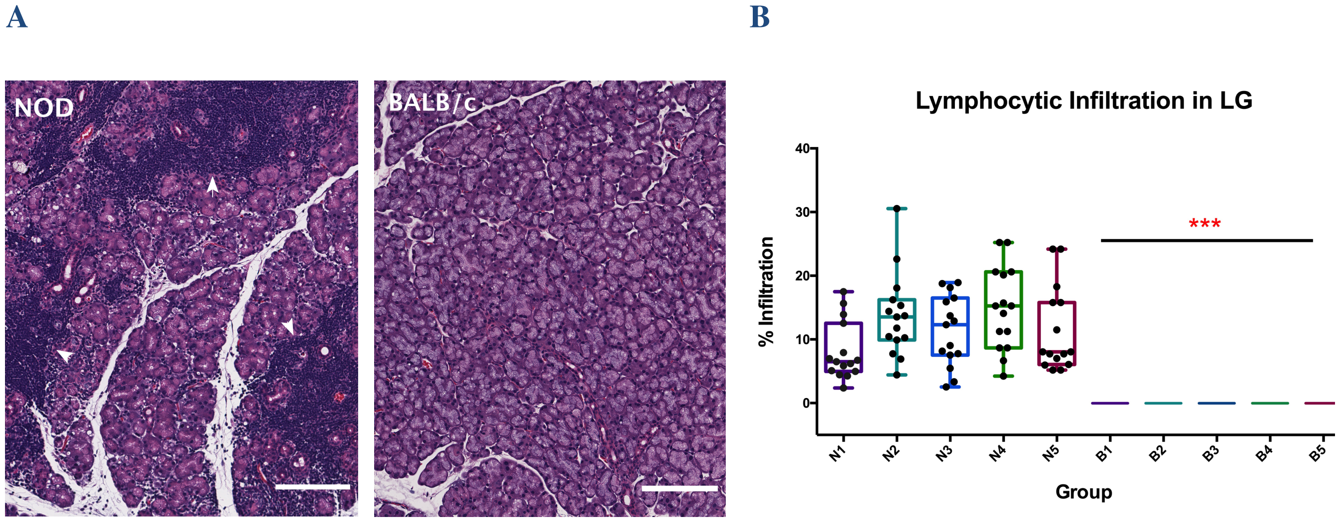

Supplementary Figure 2. Infiltration of lymphocytes in lacrimal gland (LG) of male NOD but not BALB/c mice reflects established autoimmune dacryoadenitis in the mice used as a source of exosomes. (A) Representative hematoxylin and eosin (H&E) staining of LG sections shows that lymphocytic infiltration is observed exclusively in the NOD mice (scalebar, ~250 μm). (B) Quantification of the area of infiltration in three sections per mouse LG taken at 25%, 50% and 75% depth shows that this phenotype is statistically significant in the NOD mice ($p < 0.0001$, one-way ANOVA, n =5 groups, 5 age matched mice per group)

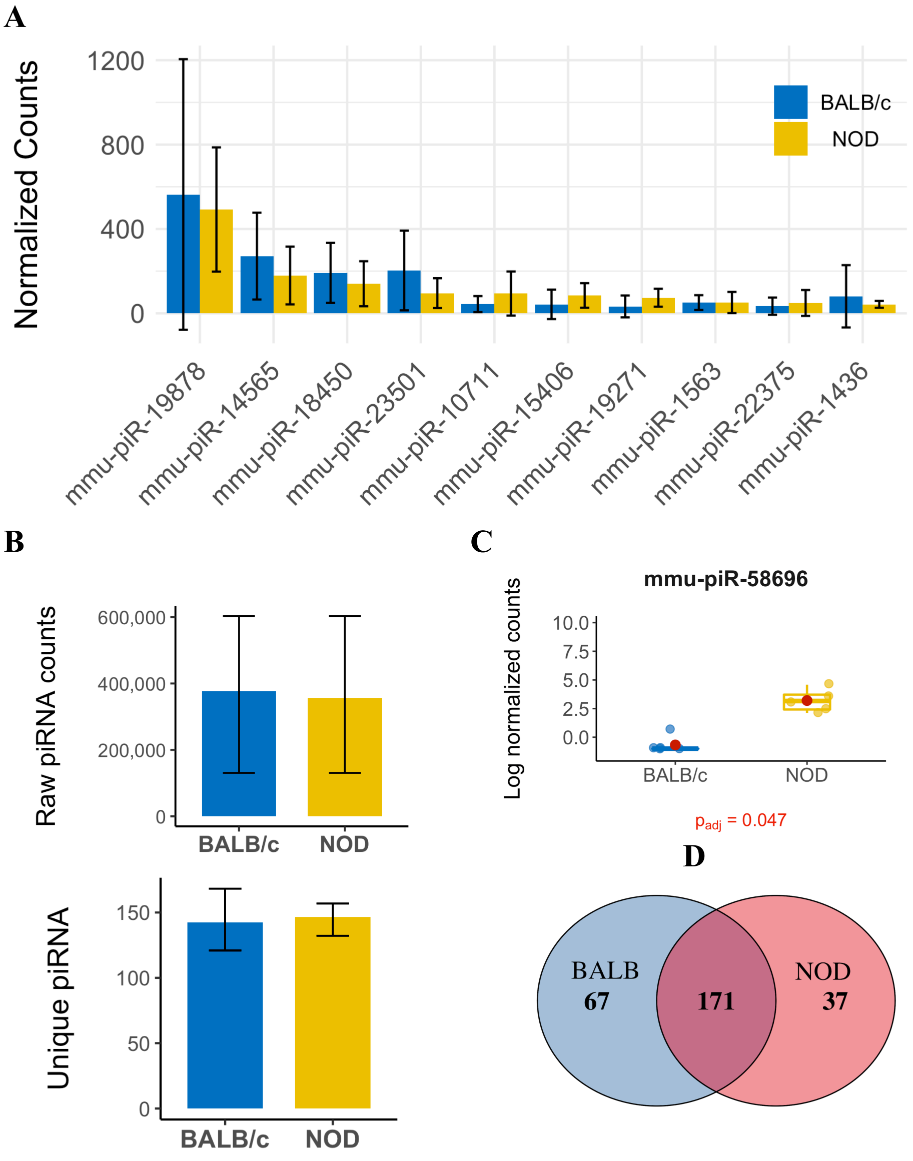

Supplementary Figure 3. Profiling of UC exosome derived piRNA & differential expression analysis. (A) Barplot comparing the top 10 highly expressed piRNA in NOD and BALB/c serum UC exosomes. (B) Barplot comparing overall expression of piRNA in NODs and BALB/c (top) and the total number of distinct piRNA expressed per strain (bottom). There was no strain specific difference in the overall expression of piRNA. (C) Differential piRNA expression analysis found that piRNA-58696 (piRdb) was significantly higher in NODs when compared to BALB/c (LimmaVoom p_{adj} = 0.047, n =5 groups, 5 age matched mice per group). (D) Venn Diagram showing number of uniquely expressed piRNA per strain.

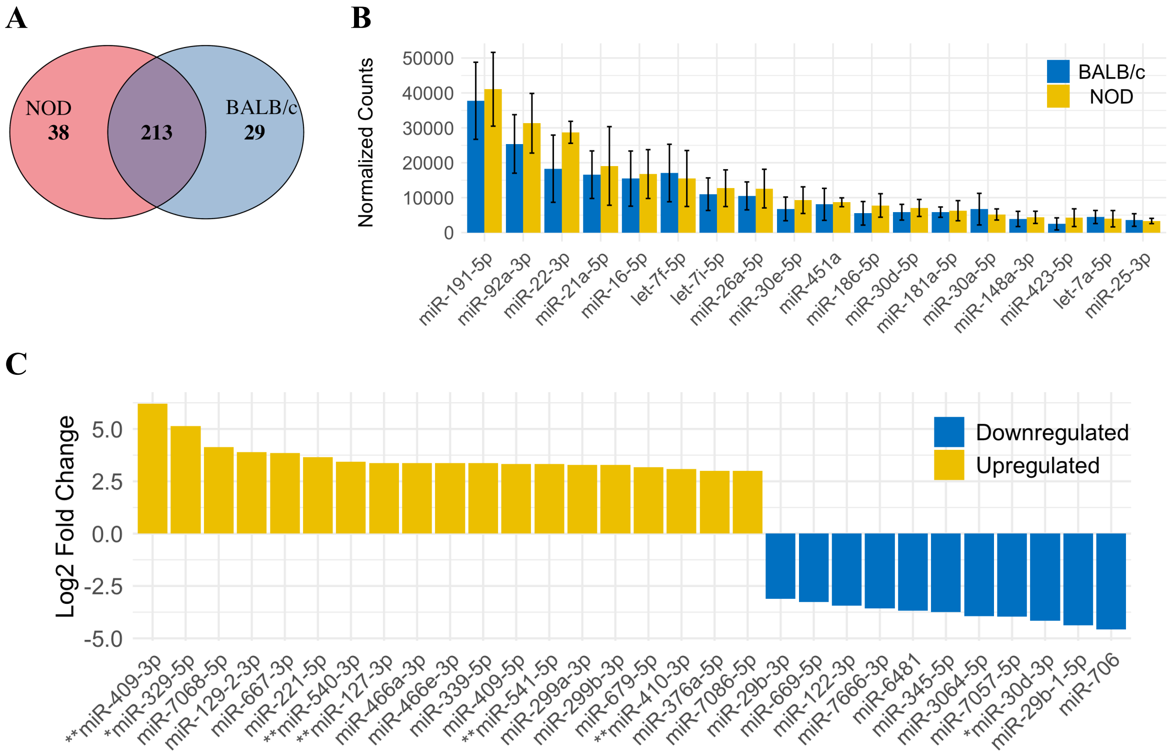

Supplementary Figure 4. Profiling of exosome derived miRNA. (A) Venn diagram showing number of detectable miRNAs per strain**. (B)** Barplot comparing the top 18 highly expressed miRNA in NOD and BALB/c UC serum exosomes from 14-week NOD and BALB/c mice. Data are plotted as raw counts normalized to total number of reads per strain that aligned to miRbase v22 (Data shown is mean ± SD, n =5 groups, 5 age matched mice per group). There was no significant difference in the expression levels of the most abundant miRNAs between the two strains. **(C)** Barplot showing miRNAs with fold change greater (or less than) 3. However, only the upregulated miRNAs reached statistical significance (** $p < 0.001$; * $p < 0.01$, DESEq2, n =5 groups, 5 age matched mice per group).

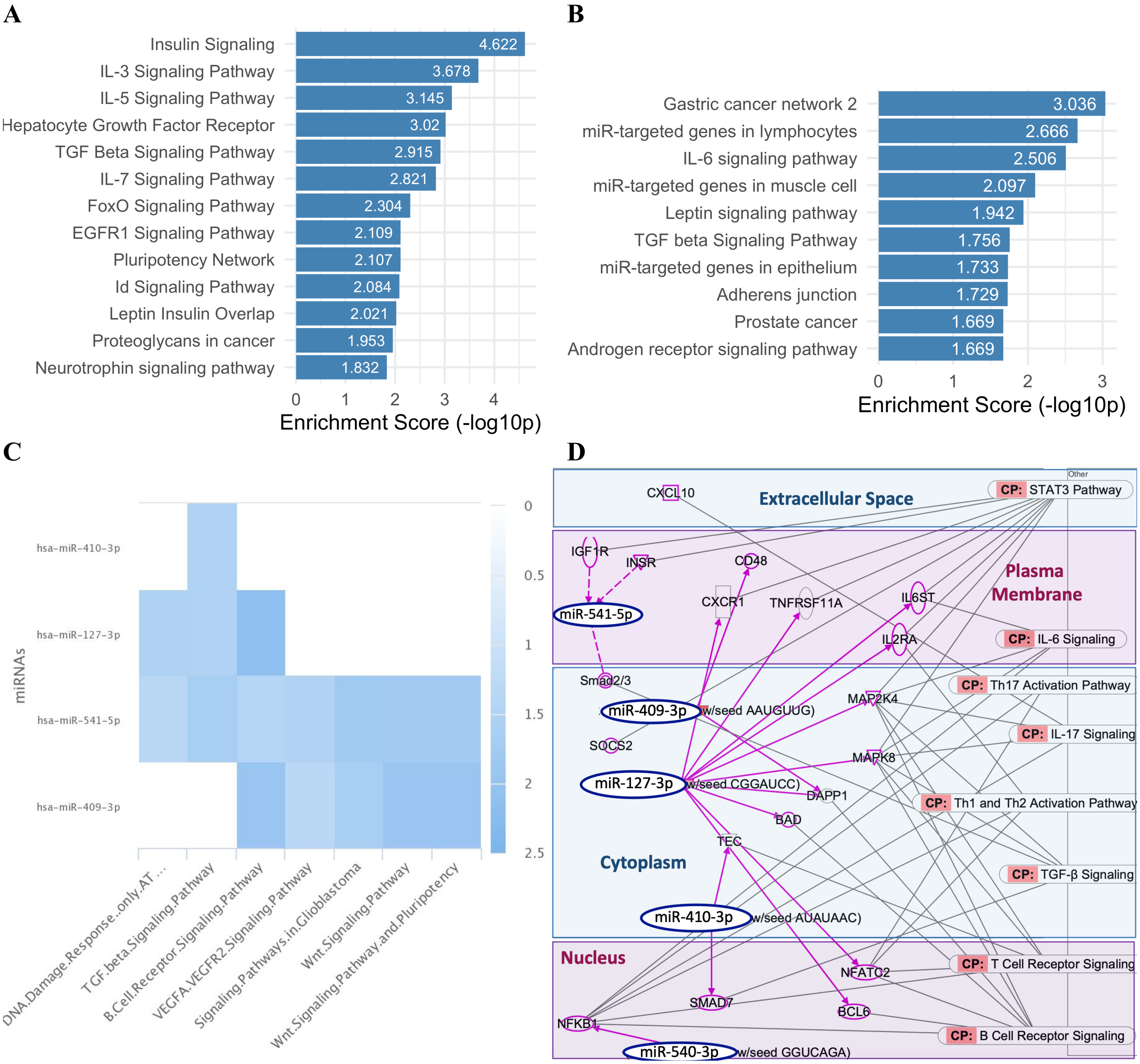

Supplementary Figure 5. Pathway analysis using miTALOS, miRPathDB and Ingenuity Pathway Analysis (IPA). Pathway analysis conducted using miTALOS for (A) mouse and (B) human miRNAs 127-3p, 409-3p and 410-3p. miRNA gene targets from experimentally validated database Starbase2 and pathways from KEGG and Wikipathways were utilized for analysis. (C) Pathway analysis conducted using miRPathDB, using experimentally validated databases. Pathways overrepresented for differentially expressed miRNA in NOD mice include those involved in cellular differentiation & proliferation (TGF-βsignaling pathway), immune regulation (IL-6, B Cell Receptor Signaling) and stem-cell maintenance. (D) IPA figure summarizing the major pathways targeted by the miRNA hits that are involved in immune regulation (including TGF-beta, IL-6 and B-cell receptor Signaling). Grey arrows indicate relationship between signal transduction pathways and pink arrows show the interaction of each specific miRNA with elements of the signal transduction pathways.

1.2 Supplementary Table

Supplementary Table 1. Reads aligned to various non-coding RNA

Sample ID	pre mirna	rRNA	lncRNA	snRNA	snoRNA	scaRNA	Mature miRNA	piRNA
**N1**	318,327	676,237	776,674	24,507	6,066	470	217,302	376,931
**N2**	1,016,375	1,622,035	1,959,601	61,225	17,458	1,170	690,950	711,308
**N3**	284,848	191,858	295,180	1,926	8,102	305	207,677	145,113
**N4**	1,671,998	488,827	765,911	20,435	12,137	1,027	1,301,840	389,208
**N5**	137,166	246,737	355,721	2,610	4,328	258	89,614	164,174
**B1**	1,765,543	863,856	1,121,746	8,662	8,567	715	1,322,082	383,467
**B2**	270,562	580,068	705,545	12,206	7,734	275	182,109	384,545
**B3**	408,015	1,316,042	1,517,483	55,577	11,547	628	286,554	787,786
**B4**	562,897	393,866	576,291	5,063	16,827	899	435,244	288,629
**B5**	422,418	191,561	329,113	1,453	1,386	86	338,684	37,871

pre-mirna – precursor microRNA; rRNA – ribosomal RNA, lnc RNA – long non-coding RNA; snRNA – small nuclear RNA; snoRNA – small nucleolar RNA, scaRNA – small cajal body specific RNA; mature miRNA – mature microRNA; piRNA – piwi interacting RNA
