# Supplementary material for: Small RNA Deep Sequencing Identifies a Unique miRNA Signature Released in Serum Exosomes in a Mouse Model of Sjögren's Syndrome
Source: Front Immunol. 2020 Jul 17;11:1475. doi: 10.3389/fimmu.2020.01475 (PMC7396589; doi:10.3389/fimmu.2020.01475)
Supplement: Supplementary file 3 [file Data_Sheet_3.pdf]

```r
setwd("~/Documents/5. TRGN 510 - Bionformatics 1/Output/")
# Creating the count Table
library(dplyr)
library(ggpubr)
library(ggsci)
library(ggplot2)
library(tidyr)
library(RColorBrewer)
library(calibrate)
library(gridExtra)
library(DESeq2)
library(DEFormats)

g1n <- read.csv('G1N_miR.csv', col.names = c("mat_miR", "N1"),
header=F)
g2n <- read.csv('G2N_miR.csv', col.names = c("mat_miR", "N2"),
header=F)
g3n <- read.csv('G3N_miR.csv', col.names = c("mat_miR", "N3"),
header=F)
g4b <- read.csv('G4B_miR.csv', col.names = c("mat_miR", "B1"),
header=F)
g5b <- read.csv('G5B_miR.csv', col.names = c("mat_miR", "B2"),
header=F)
g6b <- read.csv('G6B_miR.csv', col.names = c("mat_miR", "B3"),
header=F)
g7n <- read.csv('G7N_miR.csv', col.names = c("mat_miR", "N4"),
header=F)
g8n <- read.csv('G8N_miR.csv', col.names = c("mat_miR", "N5"),
header=F)
g9b <- read.csv('G9B_miR.csv', col.names = c("mat_miR", "B4"),
header=F)
g10b <- read.csv('G10B_miR.csv', col.names = c("mat_miR", "B5"),
header=F)
raw_count <- left_join(g1n, g2n) %>%
  left_join(g3n) %>%
  left_join(g7n) %>%
  left_join(g8n) %>%
  left_join(g4b) %>%
  left_join(g5b) %>%
  left_join(g6b) %>%
  left_join(g9b) %>%
  left_join(g10b)

rownames(raw_count) <- raw_count[,1]
raw_count <- raw_count[,-1]

#write.csv(raw_count, 'raw_count.csv')
#write(raw_count, file="raw_count.txt", sep="\t")

rm(g1n,g2n,g3n,g4b,g5b,g6b,g7n,g8n,g9b,g10b)
```

```
# Analyzing data with DESeq2
#...........................................


count_table = as.matrix(raw_count)
#rownames(count_table) <- count_table[,1]
#count_table <- count_table[,-1]

group_ = rep(c("NOD", "BALB/c"), each=5)
dge = DGEList(counts=count_table, group = group_)

#Creating the DESeq2 data object & running DESeq2
dds = as.DESeqDataSet(dge)
dds <- dds[ rowSums( counts(dds) ) > 0 , ]

dseq = DESeq(dds)
res <- results(dseq)

write.csv(res, 'GrepResults.csv')
sum(res$padj<0.05, na.rm=TRUE)

reSig <- res[which(res$pvalue < 0.005), ]
head(reSig[order(reSig$pvalue < 0.05),])
reSig$log2FoldChange[1:10]

mir_total <- colSums(raw_count[,1:10])
mir_total

colData(dseq)

res <- results(dseq, tidy=TRUE, contrast=c("group", "NOD", "BALB/c"))
%>%
  arrange(padj, pvalue) %>%
  tbl_df()

expmatrix_DESeq <- rlog(dseq, fitType="local")
expmatrix <- SummarizedExperiment::assay(expmatrix_DESeq)

library(DEGreport)

degPlot(dds = dseq, res = reSig, n = 3, xs = "group")
counts_ <- counts(dseq, normalized = TRUE)
degCheckFactors(counts_[, 6:10])

degPlotWide(dseq, genes = c('mmu-miR-409-3p', 'mmu-miR-127-3p', 'mmu-
miR-541-5p', 'mmu-miR-410-3p', 'mmu-miR-29b-3p', 'mmu-miR-196b-5p',
'mmu-let-7c-5p', 'mmu-miR-375-3p', 'mmu-miR-16-5p', 'mmu-miR-329-5p',
'mmu-miR-409-3p', 'mmu-miR-540-3p', 'mmu-miR-186-5p', 'mmu-
miR-26a-5p', 'mmu-miR-191-5p', 'mmu-miR-423-5p', 'mmu-miR-451a'),
```

```
group="group")

# Running EdgeR........................................
library(edgeR)

#1. Filtering - removing genes with zero read counts
countsPerMillion <- cpm(dge)
summary(countsPerMillion)
keep <- rowSums(cpm(dge)>1) >= 2
dge <- dge[keep, , keep.lib.sizes=FALSE]

#2. Normalization

?calcNormFactors
dge_ <- calcNormFactors(dge, method="TMM")
dge_$samples
plotMDS(dge_)

designMat <- model.matrix(~group_)
designMat

# 3. estimating Dispersions

dge_ <- estimateDisp(dge_)

et <- exactTest(dge_)

group_ <- as.factor(c("1NOD", "1NOD", "1NOD", "1NOD", "1NOD",
"2BalbC", "2BalbC", "2BalbC", "2BalbC", "2BalbC"))
group_
design <- model.matrix(~group_)

dge_ <- estimateGLMCommonDisp(dge_, design)
dge_ <- estimateGLMTrendedDisp(dge_, design)
dge_ <- estimateGLMTagwiseDisp(dge_, design)

fit <- glmQLFit(dge_, design)
qlf.2vs1 <- glmQLFTest(fit, coef=2)
topTags(qlf.2vs1)

#Using limma
Voom...................................................................
.

library(limma)

#Normalization factor calculation
dge_ <- calcNormFactors(dge)

#Filter low-expressed genes
```

```
cutoff <- 1
drop <- which(apply(cpm(dge_), 1, max) < cutoff)
d <- dge_[-drop,]
dim(dge_)
dim(d)

group_
plotMDS(dge_, col = as.numeric(group_))

#Specify the model to be fitted

mm <- model.matrix(~0 + group_)
y <- voom(dge_, mm, plot = T)

#Plot needs more filtering

#Fitting linear models

fit <- lmFit(y, mm)
head(coef(fit))

#Comparisons between groups (log fold-changes) are obtained as
contrasts of these fitted linear models. Specify which groups to
compare:
contr <- makeContrasts(group_1NOD - group_2BalbC, levels =
colnames(coef(fit)))
contr

#Estimate contrast for each gene
tmp <- contrasts.fit(fit, contr)

tmp <- eBayes(tmp)

top.table <- topTable(tmp, sort.by = "logFC", n = Inf)
head(top.table, 10)

elist <- voom(dge, design = NULL, lib.size = NULL, normalize.method =
"none", span = 0.5, plot = TRUE, save.plot = TRUE, ... = )

######################## Figures ##########################

goi <- res$row[1:8]
stopifnot(all(goi %in% names(dseq)))

poi <- res$padj[1:8]
tcounts <- t(log2((counts(dseq[goi, ], normalized=TRUE,
replaced=FALSE)+.5))) %>%
  merge(colData(dseq), ., by="row.names") %>%
  gather(gene, expression, (ncol(.)-length(goi)+1):ncol(.))
```

```
tcounts %>%
  select(Row.names, group, gene, expression) %>%
  head %>%
  knitr::kable()

tcounts$gene <- factor(tcounts$gene)

############ boxplots of the hits #############

chart_design <- theme(
  plot.title = element_text(color = "Black", size = 18, face = "bold",
margin = margin(b=25), hjust=0),
  axis.text.x = element_text(size=14),
  axis.text.y = element_text(size=13),
  axis.title.x = element_text(size=13, margin = margin(t=20),
colour="red2"),
  legend.text = element_blank(),
  legend.title = element_blank(),
  axis.title.y = element_text(size=15, margin = margin(r = 20)),
  strip.text.x = element_text(size =16, margin = margin(b=20),
face='bold', hjust=0.4),
  strip.background = element_blank(), strip.placement = "outside")

tiff("409.jpeg", units="in", width=3, height=3.5, res=600)
ggplot(tcounts[11:20,], aes(group, expression, colour=group)) +
  geom_boxplot(outlier.shape = NA, width = 0.3, coef=1.8, show.legend
= F,lwd=0.6)+
  geom_jitter(aes(fill=group), alpha =0.7, size=1.5, show.legend = F,
width=0.15)+
  labs(x=expression(paste("p"['adj'], " = 2.25 x 10" ^ '-7'  )),
       y="Log normalized counts",
       fill="Strain",
       title="") +
  theme_classic() +
  scale_colour_jco() +
  chart_design +
  stat_summary(fun.y=mean, geom="point", size=2, color="red3", alpha =
1) +
  facet_wrap(~gene, ncol=1) +
  ylim(-1.2,10.5)
dev.off()

#####changing facet scales to free x will make all the y axes better

##### Principal component analysis #######

chart_design_pca <- theme(
  axis.text.x = element_text(size=16),
  axis.text.y = element_text(size=16),
  legend.text = element_text(size=12),
```

```
  legend.title = element_text(size=14),
  axis.title.x = element_text(size=20, margin = margin(t=20)),
  axis.title.y = element_text(size=20, margin = margin(r=20)),
)

tiff("PCA1_rlog.jpeg", units="in", width=5, height=4, res=300)
plotPCA(rlog(dds), intgroup='group') +
  labs(title = " ") +
  theme_minimal() +
  chart_design_pca +
  ylim(-15,20) + xlim(-15,20) +
  geom_point(size=5, alpha=0.7) +
  scale_color_jco(name="Strain")
dev.off()

dds_ <- estimateSizeFactors(dds)
se <- SummarizedExperiment(log2(counts(dds_, normalized=TRUE) +
1),colData=colData(dds_))

tiff("PCA1_custom.jpeg", units="in", width=4, height=3, res=300)
plotPCA(DESeqTransform(se), intgroup='group') +
  labs(title = " ") +
  theme_minimal() +
  chart_design_pca +
  ylim(-15,20) + xlim(-15,20) +
  geom_point(size=8, alpha=0.9) +
  scale_color_jco(name="Strain")
dev.off()

tiff("PCA2.jpeg", units="in", width=8, height=8, res=300)
plotPCA(normTransform(dge_), intgroup='group', ntop=7) +
  labs(title = "B. PCA of top 9 hits") +
  theme_minimal() +
  chart_design_pca +
  geom_point(size=7) + ylim(-7,7) + xlim(-7,7)
dev.off()

############# Heatmap ##############

library("pheatmap")
ntd <- normTransform(dseq)
select <- order(rowMeans(counts(dseq,normalized=TRUE)),
                decreasing=TRUE)[1:150]
df <- as.data.frame(colData(dseq)[,'group'])
rownames(df) <- colnames(assay(ntd))
colnames(df) <- "strain"
tiff("hmap.jpeg", units="in", width=16, height=60, res=300)
pheatmap(assay(ntd)[select,], cluster_rows=FALSE, show_rownames=T,
         cluster_cols=T, annotation_col=df)
dev.off()
```

```
#
dds = as.DESeqDataSet(dge)
dds <- dds[ rowSums( counts(dds) ) > 0 , ]

dseq = DESeq(dds)
res <- results(dseq)

#Use unsorted res table for this
rld <- rlogTransformation(dseq)
sampleDists <- dist( t( assay(rld) ) )
as.matrix( sampleDists )[ 1:10, 1:10 ]
mat = assay(rld)[(which(res$pvalue<0.0058)),]
#mat = assay(rld)
[c(which(res$log2FoldChange>3),which(res$log2FoldChange< -4)),]
mat = mat - rowMeans(mat)
df = as.data.frame(colData(rld)[,c("group")])
colnames(df) = "Strain"
rownames(df) = colnames(mat)
my_colour = list(
  Strain=c('BALB/c'=pal_jco("default")(1), 'NOD'=pal_jco("default")(2)
[2]))
tiff("hmap.jpeg", units="in", width=4, height=3, res=300)
pheatmap(mat,
         annotation_col=df,
         color = colorRampPalette(rev(brewer.pal(n = 7, name
="RdBu")))(100),
         cutree_cols = 2,
         annotation_colors = my_colour,
         annotation_legend =F,
         angle_col=45,
         fontsize=10)
dev.off()

####### Most Abundant miRNA ######

raw_count_mini <- raw_count[which(raw_count[,1:10] > 0),]

raw_count_mini$N1 <- raw_count_mini$N1/0.217302
raw_count_mini$N2 <- raw_count_mini$N2/0.690950
raw_count_mini$N3 <- raw_count_mini$N3/0.207677
raw_count_mini$N4 <- raw_count_mini$N4/1.301840
raw_count_mini$N5 <- raw_count_mini$N5/0.089614
raw_count_mini$B1 <- raw_count_mini$B1/1.322082
raw_count_mini$B2 <- raw_count_mini$B2/0.182109
raw_count_mini$B3 <- raw_count_mini$B3/0.286554
raw_count_mini$B4 <- raw_count_mini$B4/0.435244
raw_count_mini$B5 <- raw_count_mini$B5/0.338684

raw_count_mini$NOD_mean <-
```

```
(raw_count_mini$N1+raw_count_mini$N2+raw_count_mini$N3+raw_count_mini$
N4+raw_count_mini$N5)/5
raw_count_mini$Balb_mean <-
(raw_count_mini$B1+raw_count_mini$B2+raw_count_mini$B3+raw_count_mini$
B4+raw_count_mini$B5)/5


raw_count_mini <- raw_count_mini[order(raw_count_mini$NOD_mean,
decreasing = T),]
raw_count_mini <- raw_count_mini[-246:-2751,]
raw_count_mini$SD_NOD <- apply(raw_count_mini[,1:5], 1, sd)
raw_count_mini$SD_Balb <- apply(raw_count_mini[,6:10], 1, sd)

barplot(raw_count_mini[1:20,11])

mir_abundance <- data.frame((raw_count_mini[1:20, 1:14]))
mir_abundance$miRNA <- rownames(mir_abundance)
mir_abundance$miRNA <- factor(mir_abundance$miRNA, levels =
mir_abundance$miRNA)
write.csv(mir_abundance, 'mir_abundance.csv')
MIR_abundance <- read.csv('mir_abundance.csv')

library(ggplot2)
par(cex=1.3)
tiff("NOD.jpeg", units="in", width=9, height=5, res=300)
dodge <- position_dodge(width = 0.9)
limits <- aes(ymax = mir_abundance$NOD_mean + mir_abundance$SD_NOD,
              ymin = mir_abundance$NOD_mean - mir_abundance$SD_NOD)
p <- ggplot(data = mir_abundance, aes(x = miRNA, y = NOD_mean,
fill=miRNA))
p + geom_bar(stat = "identity", position = dodge) +
  geom_errorbar(limits, position = dodge, width = 0.25) +
  theme_minimal() +
  theme(
    plot.subtitle = element_text(size=25, face="bold", hjust=1),
    axis.text.x = element_text(angle=45,hjust=1, size=15),
    axis.text.y = element_text(size=18),
    axis.title.x = element_blank(),
    axis.title.y = element_text(size=20, margin = margin(r=20)),
    legend.position = "none") +
  labs(subtitle="NOD", y = "Normalized Counts") +
  scale_y_log10()
dev.off()


par(cex=1.3)
tiff("Balb.jpeg", units="in", width=9, height=5, res=300)
dodge <- position_dodge(width = 0.9)
limits <- aes(ymax = mir_abundance$Balb_mean + mir_abundance$SD_Balb,
              ymin = mir_abundance$Balb_mean - mir_abundance$SD_Balb)
```

```
p <- ggplot(data = mir_abundance, aes(x = miRNA, y = Balb_mean,
fill=miRNA))
p + geom_bar(stat = "identity", position = dodge) +
  geom_errorbar(limits, position = dodge, width = 0.25) +
  theme_minimal() +
  theme(
    plot.subtitle = element_text(size=25, face="bold", hjust=1),
    axis.text.x = element_text(angle=45,hjust=1, size=15),
    axis.text.y = element_text(size=18),
    axis.title.x = element_blank(),
    axis.title.y = element_text(size=20, margin = margin(r=20)),
    legend.position = "none") +
  labs(subtitle="BALB/c", y = "Normalized Counts")
dev.off()

mini <- data.frame(t(raw_count_mini[order(raw_count_mini$NODsum),]))
[1:10,1:242]
mini$strain <- groups
mini$strain <- factor(mini$strain)

ggplot(data=mini, aes(x=colnames(mini[,230:240]), y=mini[1:5,])) +
  geom_boxplot()



###############most abundant miRNA Combined figure ################

raw_count_mini$NOD_mean <-
(raw_count_mini$N1+raw_count_mini$N2+raw_count_mini$N3+raw_count_mini$
N4+raw_count_mini$N5)/5
raw_count_mini$Balb_mean <-
(raw_count_mini$B1+raw_count_mini$B2+raw_count_mini$B3+raw_count_mini$
B4+raw_count_mini$B5)/5

raw_count_mini <- raw_count_mini[order(raw_count_mini$NOD_mean,
decreasing = T),]
raw_count_mini <- raw_count_mini[-142:-1442,]
raw_count_mini$SD_NOD <- apply(raw_count_mini[,1:5], 1, sd)
raw_count_mini$SD_Balb <- apply(raw_count_mini[,6:10], 1, sd)

barplot(raw_count_mini[2:20,11])

mir_abundance <- data.frame((raw_count_mini[1:20, 1:14]))
mir_abundance$miRNA <- rownames(mir_abundance)
mir_abundance$miRNA <- factor(mir_abundance$miRNA, levels =
mir_abundance$miRNA)
write.csv(mir_abundance, 'mirna_abundance.csv')
MIR_abundance <- read.csv('mirna_abundance.csv', header = T, sep=",")
rownames(MIR_abundance) <- MIR_abundance[,1]
MIR_abundance <- MIR_abundance[1:20,-1]
```

```
mir_abun <- MIR_abundance[1:20, c(11,13)]
mir_abun[21:40,] <- MIR_abundance[1:20, c(12,14)]
mir_abun[1:20,3] <- MIR_abundance[1:20,15]
mir_abun[21:40,3] <- MIR_abundance[1:20,15]
mir_abun$Strain <- rep(c("NOD", "BALB/c"), each=20)
mir_abun$Strain <- as.factor(mir_abun$Strain)
colnames(mir_abun) <- c("mean", "sd", "miRNA", "Strain")
mir_abun$miRNA <- as.factor(mir_abun$miRNA)
mir_abun$miRNA <- factor(mir_abun$miRNA,
levels=unique(mir_abun$miRNA), ordered=T)


par(cex=1.3)
tiff("Abundance_mir3.jpeg", units="in", width=8, height=3, res=300)
dodge <- position_dodge(width = 0.9)
limits <- aes(ymax = mean + sd,
              ymin = mean - sd)
ggplot(data = mir_abun[c(3:20,23:40),], aes(x = miRNA, y = mean,
fill=Strain)) +
  geom_bar(stat = "identity", position = position_dodge()) +
  geom_errorbar(limits, position = dodge, width = 0.25) +
  theme_minimal() +
  theme(
    plot.subtitle = element_text(size=16, face="bold", hjust=1),
    axis.text.x = element_text(angle=45,hjust=1, size=13),
    axis.text.y = element_text(size=13),
    axis.title.x = element_blank(),
    axis.title.y = element_text(size=14, margin = margin(r=18)),
    legend.text = element_text(size=14, hjust = 0.5),
    legend.title = element_blank(),
    legend.position = c(.9, 1),
    legend.justification = c("center", "top")) +
  labs(y = "Normalized Counts") +
  scale_fill_jco() #+
  #scale_y_log10(limits = c(1, 1000000), labels = scales::comma)
dev.off()

################### miTALOS v2.0 figure ###################

mitalos <- read.csv('miTALOS results_mice.csv')
colnames(mitalos) <- c('Source', 'Pathway', 'Enrichment_Score')
p <- ggplot(data=mitalos,aes(x=reorder(Pathway, Enrichment_Score),
y=Enrichment_Score)) + geom_bar(stat="identity", fill="steelblue")+
  theme_minimal(
    base_size = 18
  ) +
  xlab(' ') + ylab('Enrichment Score (-log10p)') +
  geom_text(aes(label=Enrichment_Score), vjust=0.5, hjust = 1.2,
color="white", size=4.5)
```

```
p + coord_flip()
par(cex=2)
tiff("mitalos_mouse.jpeg", units="in", width=6.8, height=5, res=300)
p + coord_flip()
dev.off()

mitalos_hum <- read.csv('miTALOS results_human.csv')
colnames(mitalos_hum) <- c('Source', 'Pathway', 'Enrichment_Score')
p <- ggplot(data=mitalos_hum,aes(x=reorder(Pathway, Enrichment_Score),
y=Enrichment_Score)) + geom_bar(stat="identity", fill="steelblue")+
  theme_minimal(
    base_size = 18
  ) +
  xlab(' ') + ylab('Enrichment Score (-log10p)') +
  geom_text(aes(label=Enrichment_Score), vjust=0.5, hjust = 1.2,
color="white", size=5)
p + coord_flip()
par(cex=2)
tiff("mitalos_human.jpeg", units="in", width=6.8, height=4, res=300)
p + coord_flip()
dev.off()



################# Volcano Plot ##################

#Obtain logical vector regarding whether padj values are less than
0.05
threshold_OE <- res$pvalue < 0.01
#Determine the number of TRUE values
length(which(threshold_OE))
#Add logical vector as a column (threshold) to the res table
res$threshold <- threshold_OE
#Volcano plot
ggplot(res) +
  geom_point(aes(x=log2FoldChange, y=-log10(pvalue),
colour=threshold)) +
  xlab("log2 fold change") +
  ylab("-log10 p-value") +
  theme(legend.position = "none",
        plot.title = element_text(size = rel(1.5), hjust = 0.5),
        axis.title = element_text(size = rel(1.25))) +
  theme_minimal() +
  theme(legend.title = element_blank()) +
  scale_color_discrete(name = "threshold", labels = c("NS",
"Significant"))

####### Make a basic volcano plot ######
par(cex=1.3)
tiff("Volcano2.jpeg", units="in", width=5.8, height=4.8, res=300)
```

```
with(res, plot(log2FoldChange, -log10(pvalue),
xlab=expression(paste('log'[2],'FC')), ylab=expression(paste('-
log'[10],' p value')), pch=19, xlim=c(-7.1,7.8), cex=0.65,
cex.lab=1.2, mgp=c(2.2,1,0), col="gray10"))
# Add colored points: red if padj<0.05, orange of log2FC>3
with(subset(res, abs(log2FoldChange)>3), points(log2FoldChange, -
log10(pvalue), pch=19, col="orange", cex=0.8))
with(subset(res, pvalue<0.01 & abs(log2FoldChange)>3),
points(log2FoldChange, -log10(pvalue), pch=19, col="orange", cex=1.1))
with(subset(res, pvalue<0.0005 & abs(log2FoldChange)>2.5),
points(log2FoldChange, -log10(pvalue), pch=19, col="red", cex = 1.4))
# Label points with the textxy function from the calibrate plot
with(subset(res, (padj<0.05 & abs(log2FoldChange)>2.5)) ,
textxy(log2FoldChange-0.7, -log10(pvalue)-0.5, labs=c("miR-127-3p",
"miR-409-3p", "miR-540-3p"), cex=0.7))
abline(h = 2, v = 3, col = "gray60", lty=3)
abline(h = 3.5, v = -3, col = "gray60", lty=3)
legend(-7.7, 10, legend=c(expression(paste("p"["adj"]," < 0.05 &
","log"[2],"FC < |3|")), expression(paste('log'[2],'FC', ' > |3|')),
"NS"), col=c("red", "orange","black"), pch=19, pt.cex=0.6, cex=0.6)
dev.off()

############### log2FC barplot ##################
library(ggpubr)
library(ggsci)
l2fc <- read.csv('log2fc_list.csv')
l2fc$miRNA <- factor(l2fc$miRNA, levels=l2fc$miRNA)

par(cex=1.5)
tiff("log2FC_3.jpeg", units="in", width=8, height=3, res=300)
ggplot(l2fc, aes(x=miRNA, y=log2FoldChange, fill =log2FoldChange >=
0)) + geom_bar(stat = "identity") +
  theme_minimal() +
  theme(axis.text.x = element_text(angle=45,hjust=1, size=11),
        axis.text.y = element_text(hjust=1, size=12),
        axis.title.y = element_text(size=13),
        axis.title.x = element_text(size=13),
        legend.text = element_text(size=12),
        legend.position = c(.9, .99),
        legend.justification = c("center", "top")) +
  labs(x ="", y = "Log2 Fold Change") +
  theme(legend.title = element_blank()) +
  scale_fill_jco(name = "log2FoldChange", labels = c("Downregulated",
"Upregulated"))
dev.off()


############## distribution of smRNA #######
library(scales)
```

```
theme <- theme_minimal() +
  theme(
    axis.title = element_blank(),
    panel.border = element_blank(),
    panel.grid=element_blank(),
    axis.text.x=element_blank(),
    axis.text.y = element_blank(),
    plot.title=element_text(size=13, face="bold", hjust = -0.25, color
= "#666666", vjust = 1.8),
    legend.text = element_text(size=11),
    legend.title = element_blank()
  )

smRNA <- read.csv('aligned_ncRNA.csv', header = T, row.names = 1)

NOD_av <- as.data.frame((t(smRNA[11:12,1:8])))
NOD_av$ncRNA <- factor(rownames(NOD_av))
#NOD_av$smRNA <- factor(NOD_av$smRNA, levels = NOD_av$smRNA)
NOD_av$percent <- NOD_av$NOD_av/sum(NOD_av$NOD_av)*100

NOD_av <- NOD_av %>%
  mutate(ncRNA = factor(ncRNA, levels = ncRNA)) %>%
  arrange(desc(ncRNA)) %>%
  mutate(cumenergy = cumsum(NOD_av),
         centres = cumenergy - NOD_av / 2)

par(cex=1.25)
tiff("NOD_ncRNA.jpeg", units="in", width=4.6, height=3.1, res=300)
ggplot(NOD_av, aes(x=1, y=NOD_av, fill= ncRNA)) +
  geom_bar(width =1, stat = "identity", size=0.5, colour="black") +
  coord_polar(theta = "y") +
  geom_text(x = 1.66, aes(y = centres, label = paste0(round(percent,
1), "%")), fontface="bold", size=3) +
  labs(title = "B. Mapping Overview of ncRNAs (NOD)") +
  scale_fill_jco(name=" ", guide=guide_legend(reverse = F)) +
  theme +
  scale_y_continuous(
    breaks=cumsum(NOD_av$NOD_av) - (NOD_av$NOD_av/2),
    labels=round(NOD_av$percent))
dev.off()

B_av <- as.data.frame((t(smRNA[13:14,1:8])))
#colnames(B_av) <- "counts"
B_av$smRNA <- factor(rownames(B_av))
B_av$percent <- B_av$BALB_av/sum(B_av$BALB_av)*100
B_av <- B_av %>%
  mutate(smRNA = factor(smRNA, levels = smRNA)) %>%
  arrange(desc(smRNA)) %>%
  mutate(cumenergy = cumsum(BALB_av),
         centres = cumenergy - BALB_av / 2)
```

```
par(cex=1.3)
tiff("Balb_ncRNA.jpeg", units="in", width=4.5, height=3.1, res=300)
ggplot(B_av, aes(x=1, y=BALB_av, fill= smRNA)) +
  geom_bar(width =1, stat = "identity", size=0.5, colour="black") +
  coord_polar(theta = "y") +
  geom_text(x = 1.64, aes(y = centres, label = paste0(round(percent,
1), "%")), fontface="bold", size=2.9) +
  labs(title = "C. Mapping Overview of ncRNAs (BALB/c)") +
  scale_fill_jco(name=" ", guide=guide_legend(reverse = F)) +
  theme +
  scale_y_continuous(
    breaks=cumsum(B_av$BALB_av) - (B_av$BALB_av/2),
    labels=round(B_av$BALB_av))
dev.off()

distribution <- read.csv('Read_Dist_percent.csv', header = T,
row.names = 1)
distribution$genome <- factor(rownames(distribution))

mapped = as.data.frame(distribution[,c(1,11)])
mapped[10:90,2] <- mapped$genome
mapped[10:90,1] <- c(distribution$NOD2, distribution$NOD3,
distribution$NOD4, distribution$NOD5, distribution$BALB1,
distribution$BALB2, distribution$BALB3, distribution$BALB4,
distribution$BALB5)
mapped$sample <- rep(c("N1","N2", "N3", "N4", "N5", "B1", "B2", "B3",
"B4", "B5"), each=9)
colnames(mapped) <-  c("count","genome","sample")
rownames(mapped) <- c(1:90)
mapped$sample <- factor(mapped$sample)

theme <- theme_minimal() +
  theme(
    axis.title = element_text(),
    panel.border = element_blank(),
    panel.grid=element_blank(),
    axis.text.y = element_text(size=11.5),
    axis.text.x = element_text(size=9.5),
    axis.title.x = element_text(size=12.5),
    plot.title=element_text(size=13, face="bold", hjust = 0.5, color =
"#666666", vjust = 1.8),
    legend.text = element_text(size=9),
    legend.title = element_blank(),
    legend.position = "bottom"
  )

par(cex=1.3)
tiff("Read_dist.jpeg", units="in", width=6.5, height=4, res=300)
ggplot(mapped, aes(x=sample, y=count, fill= genome)) +
```

```
  geom_bar(stat="identity") +
  coord_flip() +
  labs(title = "A. Mapping regions", y="% Mapped Reads", x="") +
  scale_fill_brewer(palette = "RdYlBu") +
  theme
dev.off()

################ venn diagram Bowtie vs miRGrep #################3

BowtieData <- read.csv('~/Documents/3_Parkinsons_disease/NGS_2018/
Linux/bowtie_run3 output/_bowtie_total_counts.csv', header=T,
row.names = 1)
BowtieData <- BowtieData[1:455,1:10]
BowtieData <- BowtieData[rowSums(BowtieData>0)>1,]
BowtieData <- rownames(BowtieData)
BowtieData <- as.list(BowtieData)

GrepData <- read.csv('raw_count.csv', row.names = 1)
GrepData <- GrepData[1:464,1:10]
GrepData <- GrepData[rowSums(GrepData>0)>1,]
GrepData <- rownames(GrepData)
GrepData <- as.list(GrepData)

library(VennDiagram)
try(library("devtools"), install.packages("devtools")) # used to
source functions from the internet
library("devtools")
source_url("http://raw.github.com/nielshanson/mp_tutorial/master/
downstream_analysis_r/code/venn_diagram2.r")
source_url("http://raw.github.com/nielshanson/mp_tutorial/master/
downstream_analysis_r/code/venn_diagram3.r")
source_url("http://raw.github.com/nielshanson/mp_tutorial/master/
downstream_analysis_r/code/venn_diagram4.r")
tiff("VennAtleast1.jpeg", units="in", width=6, height=5.3, res=300)
venn_diagram2(BowtieData, GrepData, "Bowtie", "miRGrep") +
scale_color_jco()
dev.off()


############## NOD vs BALB Venn Diagram ##########

NOD_ <- read.csv('NOD_count.csv', header=T, row.names = 1)
NOD_ <- NOD_[,c(1,2,3,4,5,11)]
NOD_ <- NOD_[rowSums(NOD_>0)>3,]
NOD_ <- rownames(NOD_)
NOD_ <- as.list(NOD_)

BALB_ <- read.csv('Balb_count.csv', header=T, row.names = 1)
BALB_ <- BALB_[,c(6,7,8,9,10,12)]
BALB_ <- BALB_[rowSums(BALB_>0)>3,]
```

```
BALB_ <- rownames(BALB_)
BALB_ <- as.list(BALB_)

library(VennDiagram)
try(library("devtools"), install.packages("devtools")) # used to
source functions from the internet
library("devtools")
source_url("http://raw.github.com/nielshanson/mp_tutorial/master/
downstream_analysis_r/code/venn_diagram2.r")
source_url("http://raw.github.com/nielshanson/mp_tutorial/master/
downstream_analysis_r/code/venn_diagram3.r")
source_url("http://raw.github.com/nielshanson/mp_tutorial/master/
downstream_analysis_r/code/venn_diagram4.r")
tiff("NvsBatleast3.jpeg", units="in", width=6, height=5.3, res=300)
venn_diagram2(NOD_, BALB_, "NOD", "BALB/c") + scale_color_jco()
dev.off()
```
